# Pesticide exposure and cortical brain activation among farmworkers in Costa Rica

**DOI:** 10.1016/j.neuro.2022.10.004

**Published:** 2022-10-10

**Authors:** Ana M. Mora, Joseph M. Baker, Carly Hyland, María G. Rodríguez-Zamora, Daniel Rojas-Valverde, Mirko S. Winkler, Philipp Staudacher, Vanessa A. Palzes, Randall Gutiérrez-Vargas, Christian Lindh, Allan L. Reiss, Brenda Eskenazi, Samuel Fuhrimann, Sharon K. Sagiv

**Affiliations:** a Center for Environmental Research and Community Health (CERCH), School of Public Health, University of California, Berkeley, 1995 University Avenue, Suite 265, Berkeley, CA 94720, USA; b Center for Interdisciplinary Brain Sciences Research, Division of Brain Sciences, Department of Psychiatry and Behavioral Sciences, School of Medicine, Stanford University, 401 Quarry Road, Stanford, CA 94305, USA; c School of Public Health and Population Science, Boise State University, 1910 W University Dr, Boise, ID 83725, USA; d Escuela de Ingenieŕia en Seguridad Laboral e Higiene Ambiental (EISLHA), Instituto Tecnológico de Costa Rica, Calle 15, Avenida 14, 1 km Sur de la Basílica de los Ángeles, Cartago 30101, Provincia de Cartago, Costa Rica; e Centro de Investigacion y Diagnóstico en Salud y Deporte, Escuela Ciencias del Movimiento Humano y Calidad de Vida, Campus Benjamin Nuñez, Universidad Nacional, Heredia 86-3000, Costa Rica; f Department of Epidemiology and Public Health, Swiss Tropical and Public Health Institute, Socinstrasse 55, 4051 Basel, Switzerland; g University of Basel, Peterspl. 1, 4001 Basel, Switzerland; h Swiss Federal Institute of Aquatic Science and Technology (EAWAG), Ueberlandstrasse 133, 8600 Dübendorf, Switzerland; i Drug and Alcohol Research Team at the Kaiser Permanente Northern California’s Division of Research, 2000 Broadway, Oakland, CA 94612, USA; j Division of Occupational and Environmental Medicine, Institute of Laboratory Medicine, Lund University, Scheelevägen 2, 22363 Lund, Sweden; k Department of Radiology, School of Medicine, Stanford University, 401 Quarry Road, Stanford, CA 94305, USA

**Keywords:** Insecticides, Farmworkers, Costa Rica, Functional neuroimaging, FNIRS

## Abstract

**Background:**

Previous epidemiological studies have reported associations of pesticide exposure with poor cognitive function and behavioral problems. However, these findings have relied primarily on neuropsychological assessments. Questions remain about the neurobiological effects of pesticide exposure, specifically where in the brain pesticides exert their effects and whether compensatory mechanisms in the brain may have masked pesticide-related associations in studies that relied purely on neuropsychological measures.

**Methods:**

We conducted a functional neuroimaging study in 48 farmworkers from Zarcero County, Costa Rica, in 2016. We measured concentrations of 13 insecticide, fungicide, or herbicide metabolites or parent compounds in urine samples collected during two study visits (approximately 3–5 weeks apart). We assessed cortical brain activation in the prefrontal cortex during tasks of working memory, attention, and cognitive flexibility using functional near-infrared spectroscopy (fNIRS). We estimated associations of pesticide exposure with cortical brain activation using multivariable linear regression models adjusted for age and education level.

**Results:**

We found that higher concentrations of insecticide metabolites were associated with reduced activation in the prefrontal cortex during a working memory task. For example, 3,5,6-trichloro-2-pyridinol (TCPy; a metabolite of the organophosphate chlorpyrifos) was associated with reduced activation in the left dorsolateral prefrontal cortex (β = −2.3; 95% CI: −3.9, −0.7 per two-fold increase in TCPy). Similarly, 3-phenoxybenzoic acid (3-PBA; a metabolite of pyrethroid insecticides) was associated with bilateral reduced activation in the dorsolateral prefrontal cortices (β = −3.1; 95% CI: −5.0, −1.2 and −2.3; 95% CI: −4.5, −0.2 per two-fold increase in 3-PBA for left and right cortices, respectively). These associations were similar, though weaker, for the attention and cognitive flexibility tasks. We observed null associations of fungicide and herbicide biomarker concentrations with cortical brain activation during the three tasks that were administered.

**Conclusion:**

Our findings suggest that organophosphate and pyrethroid insecticides may impact cortical brain activation in the prefrontal cortex – neural dynamics that could potentially underlie previously reported associations with cognitive and behavioral function. Furthermore, our study demonstrates the feasibility and utility of fNIRS in epidemiological field studies.

## 1. Introduction

Over 10 million kilograms of pesticide active ingredients are used annually in Costa Rican agriculture (Food and Agriculture Organization (FAO), 2020). Organophosphate (OP) and pyrethroid pesticides are among the most commonly applied insecticides in the country; glyphosate is the most frequently used herbicide and mancozeb the most frequently used fungicide (Vargas Castro, 2022). The application of large quantities of pesticides in agricultural fields results in elevated exposures among farmworkers and communities living near the fields. Other pesticide exposure pathways for these vulnerable groups include drift from treated fields to nearby homes, residential use, and consumption of contaminated food and water (Deziel et al., 2017; Quandt et al., 2006). Studying the health effects of pesticide exposure, particularly among highly exposed farmworkers, is of public health importance.

The epidemiologic literature to date on the neurobehavioral impact of occupational pesticide exposure has centered primarily on OP pesticides (Meyer-Baron et al., 2015; Muñoz-Quezada et al., 2016; Ohlander et al., 2020; Perry et al., 2020). Systematic reviews (Meyer-Baron et al., 2015; Muñoz-Quezada et al., 2016; Perry et al., 2020) and original studies of farmworkers from around the world (Corral et al., 2017; Wesseling et al., 2006) have linked OP pesticide exposure with poorer working memory, processing speed, and attention problems. However, despite their widespread use, there is considerably less research on the neurobehavioral effects of occupational exposure to other insecticides such as pyrethroids (Hansen et al., 2017), but also to fungicides and the herbicide glyphosate (Fuhrimann et al., 2021). Similarly, there is little data on the potential impacts of pesticide exposure on brain structure and function. Functional neuroimaging is a relatively new outcome in environmental epidemiology and could potentially illuminate mechanisms that underlie associations with neurobehavior observed in previous epidemiologic studies (Baker et al., 2017). In addition, functional neuroimaging may shed light on compensatory mechanisms that could have masked pesticide-related associations in previous studies that relied on neuropsychological measures.

To our knowledge, only two published studies of farmworkers have examined the association of pesticide exposure with brain function using neuroimaging (Bahrami et al., 2017). A study in the U.S. found brain network differences between Latino tobacco farmworkers and non-farmworkers using resting-state functional magnetic resonance imaging (fMRI) (Bahrami et al., 2017). In previous analyses using data from the same farmworkers included in the current study, we observed weak to null associations of hair and toenail manganese (Mn) concentrations – biomarkers of exposure to Mn found in dithiocarbamate fungicides used in agriculture as well as Mn in diet and drinking water – with brain activity measured using functional near-infrared spectroscopy (fNIRS) (Palzes et al., 2019). These and emergent findings from studies of children and adolescents environmentally exposed to pesticides (Binter et al., 2020, 2022; Sagiv et al., 2019) underscore the utility of functional neuroimaging for epidemiological investigations on the health effects of these chemicals (Cecil, 2022; Horton et al., 2014). Here, we employed fNIRS to examine whether exposure to a range of agricultural pesticides was associated with brain activity during three cognitive tasks designed to assess executive function, working memory, and response inhibition among farmworkers in Zarcero County, Costa Rica. We hypothesized that higher pesticide exposure levels, indicated by urinary biomarker concentrations, would be associated with alterations in cortical activation.

## 2. Methods

### 2.1. Study participants and procedures

The Pesticide Use in Tropical Settings (PESTROP) study is a cross-sectional study of 300 farmworkers from conventional and organic horticultural farms aimed at assessing the relationship between pesticide exposure, human health effects, and institutional determinants in two tropical agricultural settings: Zarcero County, Costa Rica and the Wakiso District, Uganda (Fuhrimann et al., 2019; Winkler et al., 2019). Subject recruitment and procedures of the study conducted in Zarcero County have been described in detail elsewhere (Fuhrimann et al., 2019; Palzes et al., 2019; Staudacher et al., 2020). Briefly, a total of 200 Global Positioning System (GPS) points were randomly generated based on smallholder land-use data (Weiss, 2021). Research assistants visited these GPS points and determined which ones were active horticultural farms after meeting with farm owners or administrators. When the GPS point did not correspond to an active farm, the closest smallholder farm within a radius of 1 km was registered; if no farm was nearby, the GPS point was dropped. Certified organic farms were identified from a list provided by the organic farmers’ association or through onsite identification.

After conventional and organic farms were identified, farm owners were briefly informed about the PESTROP study aims and procedures. If they were interested in the study, basic contact information was collected to schedule a later visit to their farms to meet with their farmworkers and invite them to participate. Eligible farmworkers were age 18 years or older, owned or worked on a conventional or organic farm within the study area, and had no self-reported diagnosis of a psychiatric disorder or use of psychopharmacologic medications. For the current study, we selected a convenience subsample of 48 (16%) out of the 300 PESTROP study participants due to limitations on the availability of fNIRS equipment and technical staff. We recruited a roughly equal distribution of organic (*n* = 26; 54%) and conventional (*n* = 22; 46%) farmworkers to ensure that there was sufficient variability in their pesticide exposure.

We assessed study participants over two visits conducted between July and August 2016. Study visits took place at the farms where they worked or at their homes. During the first visit, trained research assistants administered participants a structured questionnaire to collect data on socio-demographic characteristics (e.g., age, country of birth, education level, marital status, family income), occupational and medical history (e.g., age when started working in agriculture, age at first contact with pesticides, diagnosis of any illness), pesticide use (e.g., any pesticide applications during the last 12 months and the last week), and computer literacy (i.e., “Have you ever used a computer or played video games?”). Following the questionnaire, participants underwent the fNIRS scan and provided a urine sample. The follow-up study visit occurred approximately one month later [mean (SD) = 29.7 (2.7) days] and included the administration of a short questionnaire on recent pesticide exposure (e.g., any pesticide applications during the last week) and the collection of a second urine sample. The Human Subjects Committee of the Universidad Nacional in Costa Rica (UNA-CECUNA-ACUE-04-2016) and the Ethical Board of the Ethikkommission Nordwest-und Zentralschweiz in Switzerland (EKNZ-UBE 2016-00771) approved all study materials and procedures. Written informed consent was obtained from study participants at enrollment.

### 2.2. Urinary pesticide measurements

Spot urine samples were collected after handwashing and stored in 100 mL single-use sterile polypropylene containers (Vacuette^®^, sterile) at 4 °C until the end of the fieldwork day. Study staff aliquoted samples into 15-mL test tubes (PerformRTM Centrifuge tubes, Labcon^®^, sterile) and stored them at − 20 °C until shipment to Lund University, Sweden for analysis. Research assistants used disposable nitrile gloves when handling the urine samples. Equipment was triple rinsed with water and work surfaces were cleaned with a disinfectant before and after handling urine samples.

Urine samples were analyzed for 13 pesticide biomarkers (see Table S1), including five insecticide metabolites [3,5,6-trichloro-2-pyridinol (TCPy; a metabolite of the OP chlorpyrifos) and four metabolites of pyrethroid insecticides: 3-phenoxybenzoic acid (3PBA), 4-fluoro-3-phenoxybenzoic acid (4F3PBA), sum of cis/trans 3-(2,2-dichlorovinyl)– 2,2-dimethylcyclopropanecarboxylic acid (DCCA), and chloro-3,3,3-trifluoro-1-propene-1-yl (CFCA)]; five fungicide metabolites [ethylenethiourea (ETU; a metabolite of mancozeb and maneb), propylenethiourea (PTU; a metabolite of propineb), 5-hydroxy-thiabendazole (OH-T; a metabolite of thiabendazole), 3-hydroxy-pyrimethanil (OH-P; a metabolite of pyrimethanil), and t-butyl-hydroxy tebuconazole (TEB-OH; a metabolite of tebuconazole)]; and three herbicides or herbicide metabolites [2,4-dichlorophenoxyacetic acid (2,4-D; parent compound), glyphosate (GLY; parent compound), and aminomethylphosphonic acid (AMPA; a degration production of glyphosate in the environment)]. We analyzed all urine samples using methods described previously (Norén et al., 2020). Briefly, for the analysis of TCPy, 3PBA, 4F3PBA, DCCA, OH-T, OH-P, TEB-OH, and 2,4-D, samples were de-conjugated using β-glucuronidase/arylsulfatase and extracted using solid phase extraction (SPE). For ETU and PTU measurements, urine samples were hydrolyzed using a basic buffer. For GLY and AMPA measurements, urine was diluted using an acid buffer prior to analysis. We conducted quantitative analysis using liquid chromatography-triple quadrupole linear ion trap mass spectrometry (LC/MS/MS; QTRAP 5500 or 6500 +; AB Sciex, Framingham, MA, USA). All batches included laboratory blanks and in-house quality control samples (QC). Between-run precisions of QCs were 3–22%. The limits of detection (LOD) were defined from the chemical blanks and are shown in Table 2. The laboratory at Lund University takes part in the Erlangen inter-laboratory program for TCPy 3-PBA, and GLY with excellent results (see certificate of participation in Supplementary Material).

Urinary specific gravity (kg/L) was determined using a hand refractometer and pesticide biomarker concentrations were normalized for dilution using the formula *P*_*SG*_ = *P* × [(1.017 − 1)/(*SG* − 1)], where *P*_*SG*_ is the specific gravity-corrected pesticide biomarker concentration (μg/L), *P* is the observed pesticide biomarker concentration (μg/L), *SG* is the specific gravity of the urine sample, and 1.017 kg/L is the average specific gravity for our study population.

We decided a priori to include in our analyses only pesticide metabolites or parent compounds with a detection frequency of 65% or more at both study visits (i.e., TCPy, 3PBA, DCCA, ETU, PTU, TEB-OH, 2,4-D, GLY) (Lubin et al., 2004). We imputed concentrations below the LOD only for the selected compounds using robust regression on order statistics (Helsel, 2012).

### 2.3. fNIRS data collection and preprocessing

**Fig. 1.**
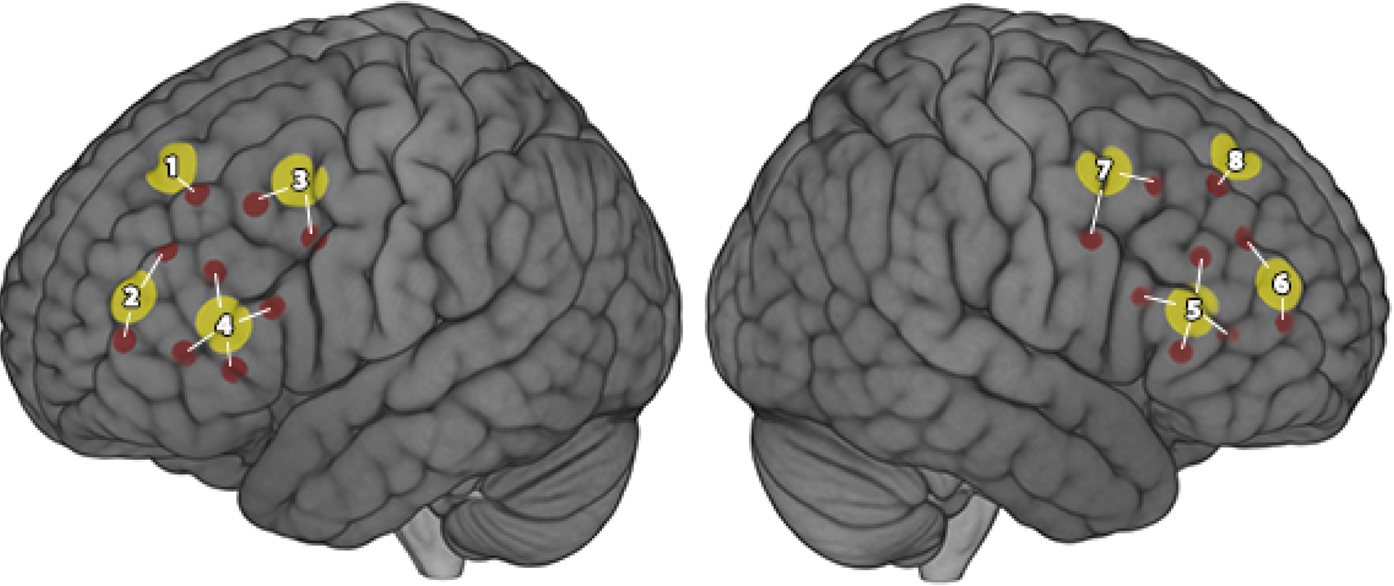
FNIRS source arrangement and channel clustering used in our study of farmworkers from the Zarcero County, Costa Rica. Red circles represent a channel (source and detector pair). Yellow circles are clusters based on proximity of channels and anatomy and include Source cluster (Sc) 1: Left superior frontal pole; Sc2: Left inferior frontal pole; Sc3: Left dorsolateral prefrontal cortex; Sc4: Left Broca’s/Broadmann Area 44/45; Sc5: Right Broca’s/BA 44/45; Sc6: Right inferior frontal pole; Sc7: Right dorsolateral prefrontal cortex; Sc8: Right superior frontal pole.

We used fNIRS, an optical neuroimaging technology that measures hemodynamic changes in the cerebral cortex (i.e., outermost layer of the brain, just beneath the scalp), to assess cortical neural activation. Details of the fNIRS methods used in this study have been described previously (Baker et al., 2017; Palzes et al., 2019; Sagiv et al., 2019). Briefly, we used a NIRSport (NIRx Medical Technologies, Germany) device outfitted with eight source and eight detector optodes (Fig. 1) to project near-infrared light with wavelengths of 760 nm and 850 nm and sampled at a rate of 7.81 Hz. Participants were fitted with appropriately sized elastic brain imaging caps (Brain Products, Germany) based on their head circumference. Optodes were affixed to the caps using pre-determined International 10/20 locations (Okamoto et al., 2004; Tsuzuki et al., 2012), resulting in 18 channels configured to assess hemodynamic fluctuations within the bilateral prefrontal cortex (Okamoto et al., 2004; Tsuzuki et al., 2012). We achieved consistent 3-cm recording channels between each source/detector pair using plastic supports bilaterally across the prefrontal cortex, the brain region that underlies most of our neurobehavioral domains of interest (i.e., working memory, attention, and cognitive flexibility).

Study staff placed the optode-fitted cap on the participant’s head, performed calibration tests, and adjusted optodes as needed. Participants completed three computer-based tasks on a laptop computer during the fNIRS scan: (i) the Sternberg working memory test; (ii) the Go/No-Go test; and (iii) the Wisconsin Card Sort Test (WCST). We presented the three tasks in that same order to all study participants. We selected these tests because they are related to neurobehavioral functions that had been previously linked with pesticide exposure in occupational and non-occupational studies, including working memory, attention, and cognitive flexibility (Kori et al., 2018; Meyer-Baron et al., 2015; Muñoz-Quezada et al., 2016; Rohlman et al., 2014; Ross et al., 2013; Sagiv et al., 2021; Wesseling et al., 2006).

The Sternberg is a test of letter-retrieval working memory, in which participants are asked to memorize a list of seven or eight letters displayed for 2 s (Encoding phase), hold those letters in their memory (Maintenance phase), and then recall whether a single letter was part of the previous list or not (Recall phase). During the Recall phase, participants press a button on the keyboard to indicate their response (yes/no), and reaction time and accuracy are recorded. The task consists of 30 trials with a jittered Inter-trial interval (ITI) (4 s) where participants passively view a fixation cross. Participants are asked to relax, remain still, and look at the fixation cross for 30 s at the beginning and end of the task (Rest). The Go/No-Go is a test of attention and response inhibition, in which participants are instructed to press a button when any letter other than ‘X′ appears (i.e., Go trials), and withhold pressing the button when ‘X′ was shown (i.e., No-Go trials). The task consisted of two alternating conditions (Go and No-Go), in which a letter was presented every 2 s (500 ms stimulus, 1500 ms inter-stimulus interval) in the middle of the computer screen, and a response was made via a key press. In the Go (control) condition, participants were presented with a random sequence of letters other than the letter “X”. In the NoGo (experimental) condition, participants were presented with the letter “X” on half (50%) of all trials (Cui et al., 2011). The WCST is a test of cognitive flexibility and executive function, in which participants are asked to match cards based on an unstated rule. Participants are presented four fixed reference cards situated evenly along the bottom half of the computer screen, each containing a different configuration of geometric figures (dot, star, cross, or triangle), numbers (1–4), or colors (red, blue, yellow, or green). The configuration of each reference card is fixed, and does not vary across participants. On each ‘match’ trial, a new test card is presented in the top center portion of the screen, and participants match the test card to a reference card based on an unstated criterion (i.e., matching on shape, number, or color). In this condition, the test card is never a perfect match with any reference card configuration. Participants receive auditory feedback (right or wrong) through which they deduce the sorting rule. Participants are given 15 trials per block to identify and correctly respond to the rule six times in a row, and the rule is pseudo-randomly changed after each block. In the control (exact match) condition, the test card is identical to one of the four fixed reference cards and the participant indicates the card with the exact match. Furthermore, in this condition participants have to correctly respond to the exact match sorting rule eight times in a row.

We developed and presented all tasks using the Psychophysics Toolbox (Kleiner et al., 2007) in MATLAB. Event markers required for fNIRS data analysis were entered into the data stream in real time during each scan. We preprocessed all fNIRS data following the pipeline outlined by Brigadoi and colleagues (Brigadoi et al., 2014) and using the Homer2 fNIRS analysis package (https://homer-fnirs.org/) (Cui et al., 2010, 2011; Huppert et al., 2009). First, raw data were converted to optical density using a partial pathlength factor of 6.06, and were corrected for motion-related artifacts using a wavelet-based correction procedure (Hosseini et al., 2017). Second, data were band-pass filtered between 0.01 Hz and 0.5 Hz (Cui et al., 2010). Quality of filtered data was assessed using the Homer2 ‘enPruneChannels’ function, as well as the correlation-based method described by Cui and colleagues (Cui et al., 2010). Any data channel that was flagged by both methods (6.78%) was removed from downstream analyses. However, in no case were all channels in a source cluster rejected within a given participant. Third, the preprocessed data were converted into time series of oxygenated hemoglobin (HbO) and deoxygenated hemoglobin (HbR) concentrations using the modified Beers-Lambert law (Wyatt et al., 1986).

We fitted generalized linear models (GLMs), using MATLAB 2012b code written in-house (Baker et al., 2016, 2018, 2020; Bruno et al., 2018), to assess patterns of cortical activation that occurred in response to task-specific cognitive demands. Our GLM procedure assumed a Gaussian hemodynamic response function. The onset and duration of each condition of interest were used as predictor variables in our GLMs to estimate standardized beta (β) coefficients for each condition and within each channel. The sign and magnitude of each β coefficient provides an indicator of the direction (negative/positive) and intensity of change in hemoglobin oxygenation (i.e., cortical brain activity) that occurred during each condition. We estimated β coefficients for all task and control conditions: the ‘Encoding’, ‘Maintenance’, ‘Retrieval’, and ‘ITI’ portions of the Sternberg test; the ‘No-Go’, ‘Go’, and ‘ITI’ portions of the Go/No-Go test; and the ‘Matching’ blocks, ‘Control’ blocks, and ‘Inter-Block Interval’ (IBI) portions of the WCST. In order to capture the cortical activation unique to the task demands, and thus not expected to be present in signals corresponding to the control conditions, we computed contrasts between the coefficients estimated for each condition and their respective control: 1) Encoding vs. Recall for the Sternberg working memory task; 2) No-Go vs. Go for the Go/No-Go task; and 3) Matching vs. Control for the WCST.

We used a functional localization approach (Baker et al., 2018; Bruno et al., 2018; Hosseini et al., 2017) to account for variation in cortical activation in response to our tasks. This approach allows for minor variation in the location of task-responsive brain regions across participants and reduces the risk of committing type II (i.e., false negative) errors that occur when averaging across nonresponsive channels. We grouped channels based on proximity and anatomical location (Fig. 1) to create eight clusters or functional regions of interest (ROIs). Within each of the eight ROIs, we selected and submitted for group-level analysis the channel with the greatest contrast value. We conducted the localization procedure first on the HbO data, then selected the same eight channels for the HbR data. We used one-sample t-tests to determine if each group-level localized contrast differed significantly from zero and the False Discovery Rate (FDR) procedure to correct for inflated risk of Type I errors due to multiple testing (Benjamini and Hochberg, 1995).

We excluded data due to technical issues with data collection (i.e., failure of study participant to complete the task, failure of the task computer to present the task, failure of the task computer to record the performance data, or failure of the fNIRS device to record the fNIRS data correctly), and data cleaning (i.e., low correlation between HbO and HbR, change in signal to noise ratio measured by the Homer2 ‘enPruneChannels’ function, or critically low signal quality based on NIRx calibration methods). These exclusions reduced our sample size from 48 to 41 participants for the WCST only.

### 2.4. Statistical analyses

We generated plots and descriptive statistics for all variables, and bivariate associations between exposure biomarkers, outcomes, and covariates using t-tests for continuous variables and χ^2^ tests for categorical variables. We estimated correlations between specific gravity-corrected urinary pesticide biomarker concentrations using Spearman’s correlation coefficients (r_s_). To assess the within- and between-worker variability and reproducibility of pesticide biomarker concentrations, we calculated intraclass correlation coefficients (ICCs) using mixed-effects models (McGraw and Wong, 1996).

We averaged specific gravity-corrected urinary pesticide biomarker concentrations across the two samples collected for each farmworker and log_2_-transformed mean concentrations to reduce the influence of extreme values (if a participant had only one measure, we used that value). We fitted linear regression models to estimate associations (β and 95% CI) of urinary pesticide biomarkers with brain activation, as indicated by HbO concentrations, for each of the three fNIRS contrasts described above [i.e., Encoding vs. Recall (Sternberg working memory task); 2) No-Go vs. Go (Go/No-Go task); and 3) Matching vs. Control (WCST)]. β coefficients represent the change in brain activation during a challenge vs. control task per two-fold increase in specific gravity-corrected urinary pesticide biomarker concentrations. We adjusted our models for age (continuous) and education level (<6th grade, 7th-11th grade), both strong predictors of neurobehavioral outcomes (Kori et al., 2018; Meyer-Baron et al., 2015; Muñoz-Quezada et al., 2016; Rohlman et al., 2014; Ross et al., 2013; Wesseling et al., 2006). We examined exposure-outcome associations both controlling for type I error using the Benjamini-Hochberg FDR (Benjamini and Hochberg, 1995) at < 0.05 and without correcting for multiple comparisons. We tested for linearity of our exposure-outcome associations using generalized additive models (GAMs) with three degrees of freedom cubic splines. Since linearity was justified across most associations (see examples in Fig. S2), we included pesticide biomarker concentrations parameterized as continuous variables in all models.

In secondary analysis, we used two-stage Bayesian Hierarchical Models (BHM) to examine exposure-outcome associations with all urinary pesticide biomarkers included simultaneously while dealing with issues of collinearity and multiple comparisons (Greenland, 1994, 2000; Greenland and Poole, 1994; MacLehose et al., 2007; MacLehose and Hamra, 2014; Rothman et al., 2012). In the first stage, we regressed brain activation for each of the three fNIRS contrasts on the pesticide exposures and covariates in single linear models. In the second stage, we modeled regression coefficients from the first stage (β) with linear weighted-least squares regression models that are a function of the regression coefficient vectors and residual errors. We specified vague priors on some model parameters and prespecified the variance for the residual error based on results from the single linear regression models and prior experience with these exposures. We selected a variance (τ) that assumed that β parameters would lie between − 3.0–3.0. We present β and 95% credible intervals (CrI) for each pesticide biomarker predicted from the second-stage model.

In addition to estimating associations of pesticide exposure with brain activation from fNIRS, we examined associations of urinary pesticide biomarker concentrations with performance on tasks administered during fNIRS, including accuracy, errors, and reaction time. We also conducted sensitivity analyses to assess the robustness of our results. These included fitting single-pollutant linear regression models for each of the three fNIRS tasks using HbR concentrations instead of HbO concentrations; but also models with HbO concentrations that excluded left-handed participants (*n* = 3), female participants (*n* = 2), participants with self-reported neurological disorders (i.e., epilepsy; *n* = 1), and participants who had outliers in task performance measures [outliers were defined as *x* < [P25 – 1.5 * (P75-P25)] and/or *x* > [P75 + 1.5 * (P75-P25)] (Rosner, 2015); *n* = 1–4, depending on the performance measure]. Lastly, we adjusted our single-pollutant linear regression models for additional potential confounders or strong predictors of the outcome [i.e., poverty status (< poverty line, > poverty line) and computer literacy (dichotomous)]. We used the R missForest package to impute missing values for poverty status (6% missing; included type of farm, time working in agriculture, and education level in the random forest model) and computer literacy (8% missing; included education level and poverty status in the model). We conducted all analyses using R version 4.1.2 (R Foundation for Statistical Computing, Vienna, Austria).

## 3. Results

As shown in Table 1, farmworkers in the fNIRS study sample (*n* = 48) were predominantly male (96%), had a low educational level (65% had <6th grade education), and most were living above the poverty line (67%). Median (P25-P75) age at assessment and time handling pesticides were 31 (24–52) and 20 (10–33) years, respectively. Participants in the fNIRS substudy were more likely to be born in Costa Rica (71%), to consume any alcohol at the time of enrollment (94%), and to have ever used a computer or played videogames (56%), compared with non-participants in the larger Costa Rica PESTROP study (57%, 69%, and 32%, respectively) (Table 1).

### 3.1. Pesticide biomarker concentrations

Detection frequencies for the 13 pesticide biomarkers ranged from 23% to 100% (Table 2 and S2), with eight of the biomarkers having a detection frequency of 65% or more in urine samples collected at both study visits (i.e., TCPy, 3-PBA, DCCA, ETU, PTU, TEB-OH, 2,4-D, and GLY). Out of these eight pesticide biomarkers, six had concentrations that varied more between than within workers (ICC > 0.50; Table 2); urinary concentrations of TEB-OH and 2,4-D varied more within than between workers (ICC = 0.38 and 0.21, respectively). Correlations between repeated measurements of urinary pesticide biomarkers ranged between − 0.12 for 2,4-D and 0.81 for TCPy (Table S3). Geometric mean (GM) [geometric standard deviation (GSD)] specific gravity-adjusted urinary TCPy, 3-PBA, ETU, and GLY concentrations averaged over the two study visits were 8.6 ng/mL (3.1), 1.5 ng/mL (2.5), 1.2 ng/mL (3.0), and 0.4 ng/mL (2.3), respectively (Table 2). Correlations between averaged urinary pesticide biomarkers varied extensively (r_s_ = −0.16 to 0.92; Fig. S1) but were the highest between the pyrethroid metabolites 3-PBA and DCCA (r_s_ = 0.92), and between glyphosate and its metabolite AMPA (r_s_ = 0.54).

Study participants working in conventional farms had higher concentrations of insecticide and fungicide metabolites in urine compared to those working in organic farms [e.g., GM (GSD) specific gravity-adjusted urinary TCPy concentrations = 17.1 ng/mL (2.9) vs. 4.8 ng/mL (2.3), respectively; Table S4]. Farmworkers living at or below the poverty line had higher urinary concentrations of ETU and PTU [GM (GSD) = 2.0 ng/mL (3.7) and 0.6 ng/mL (3.3), respectively] compared to those living above the poverty line [GM (GSD) = 0.9 ng/mL (2.5) and 0.3 ng/mL (2.9), respectively; Table S4]. Participants who applied any pesticides during the week before the study visit had higher concentrations of insecticide metabolites compared to those who did not apply pesticides [e.g., GM (GSD) TCPy concentrations = 10.4 ng/mL (3.4) vs. 5.6 ng/mL (2.0), respectively; Table S4].

### 3.2. Cortical brain activation

We observed significantly greater cortical brain activity during the test vs. control condition for each of the three tests that we administered. As shown in Fig. S3, for all ROIs, bilateral cortical brain activation was higher during the Encoding vs. Maintenance condition of the Sternberg working memory task (Fig. S3A) and the No-Go vs. Go condition for the Go/No-Go task (Fig. S3B). For the WCST, cortical activity was higher for the Matching vs. Control condition only in the bilateral prefrontal cortices (Fig. S3C).

### 3.3. Associations of pesticide biomarker concentrations with cortical brain activation

**Fig. 2.**
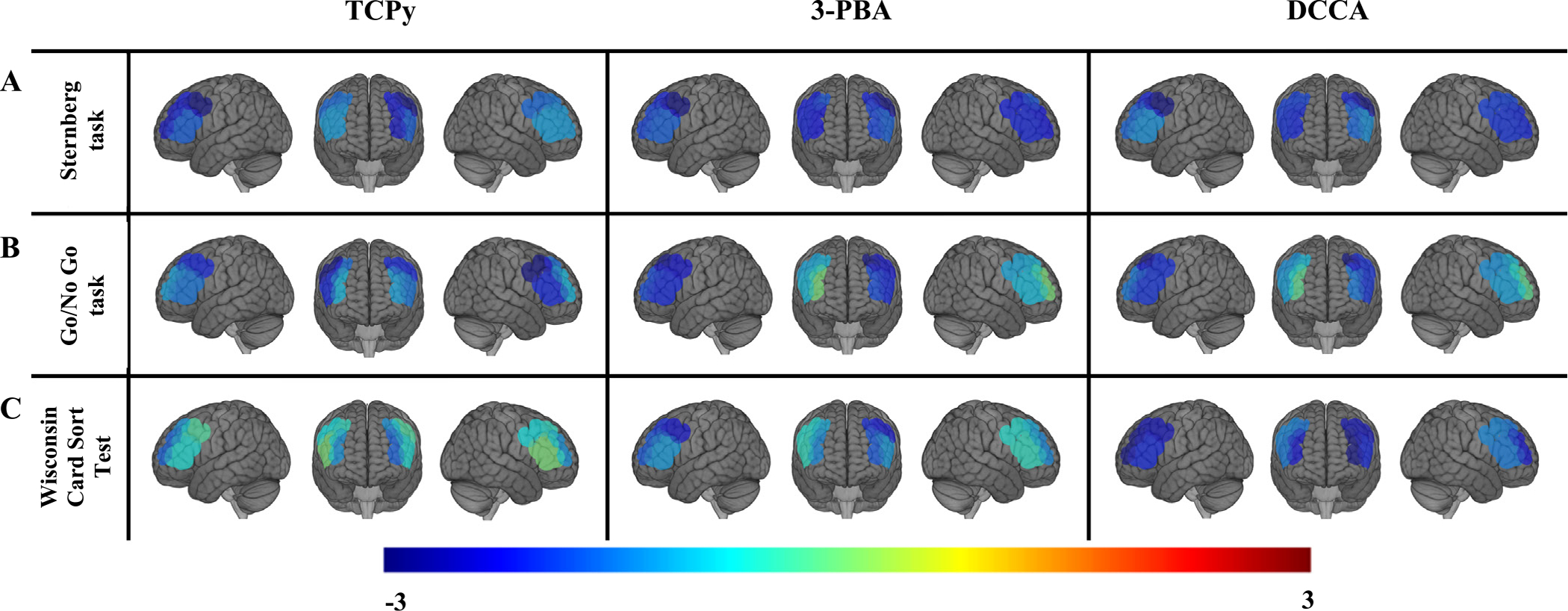
Regions with significant (non-FDR corrected p-value <0.05) associations of urinary organophosphate and pyrethroid insecticide metabolite concentrations with cortical brain activation (reduced activation) during the (A) Sternberg test (*n* = 48), (B) Go/No-Go test (*n* = 48), and (C) Wisconsin Card Sorting Test (*n* = 41) in farmworkers from the Zarcero County, Costa Rica. Models adjusted for age (continuous variable) and education level (≤6th grade, 7–11th grade). *Abbreviations*: DCCA, 3-(2,2-dichlorovinyl)–2,2-dimethylcyclopropanecarboxylic acid; TCPy, 3,5,6-trichloro-2-pyridinol; 3-PBA, 3-phenoxybenzoic acid.

We observed that higher urinary TCPy concentrations were associated with reduced brain activation, as indicated by HbO concentrations, in the prefrontal cortex of the left hemisphere during the Sternberg test (Table 3 and Fig. 2A), particularly in the left dorsolateral prefrontal region [covariate-adjusted but not false discovery rate (FDR)-corrected β per two-fold increase in TCPy concentrations = −2.3; 95% CI: − 3.9, − 0.7]. Higher urinary 3-PBA and DCCA concentrations were also associated with reduced brain activation bilaterally in the prefrontal cortex during the Sternberg working memory test (Table 3, Figs. 2B and 2C), with the stronger associations for the dorsolateral prefrontal regions (e.g., β per two-fold increase in 3-PBA concentrations = −3.1; 95% CI: −5.0, −1.2 and −2.3; 95% CI: −4.5, −0.2 for left and right cortices, respectively) and superior frontal lobes (e.g., β per two-fold increase in 3-PBA concentrations = −2.3; 95% CI: −4.1, −0.5 and −1.9; 95% CI: −3.8, −0.1 for left and right lobes, respectively). Although considerably fewer observed associations were statistically significant after correcting for multiple comparisons, associations of TCPy, 3-PBA, and DCCA concentrations with reduced cortical activation in the left dorsolateral prefrontal region remained statically significant.

Associations were weaker and not statistically significant for urinary 3-PBA and DCCA concentrations and cortical activation during the Go/No-Go and WCST tasks, but patterns of reduced activation in the prefrontal cortex of the left hemisphere were similar to those observed during the Sterberg test (Table 3, Figs. 2B and 2C). We found null associations between urinary TCPy concentrations and brain activation for the Go/No-Go and WCST tasks (Table 3, Figs. 2B and 2C). We also observed mostly null associations of fungicides and herbicides with cortical brain activation across all tasks and ROIs (Table 3).

### 3.4. Secondary analyses

BHM estimates were generally similar in direction to the single-pollutant linear regression models estimates (Table S5). However, most exposure-outcome associations were attenuated, except for the associations of urinary GLY concentrations with reduced brain activation in the left dorsolateral prefrontal region (β per two-fold increase in GLY concentrations = −3.0; 95% CI: −5.5, −0.5) and the right superior frontal lobe (β = −3.5; 95% CI: −6.2, −0.8) during the WCST task which became stronger but also less precise (Table S5).

### 3.5. Sensitivity analyses

There were no notable patterns of associations between urinary pesticide biomarkers and HbR concentrations (Table S6). We also observed mostly null associations between urinary pesticide biomarkers concentrations and performance (e.g., accuracy, errors, and reaction time) on the tasks administered with the fNIRS (Table S7). We did not find any material differences in pesticide biomarkers concentrations and brain activation associations when we restricted our analyses to right-handed individuals (Table S8), male participants (Table S9), participants without neurologic disorders (Table S10), or participants without outliers in task performance measures (Tables S11–S14). Effect estimates were also unchanged when we adjusted our models for poverty status (Table S15) and computer literacy (Table S16).

## 4. Discussion

In this study, we observed consistent negative associations between insecticide metabolite concentrations, including TCPy (metabolite of the OP chlorpyrifos) and 3-PBA and DCCA (metabolites of pyrethroid insecticides), and cortical activation across regions of the prefrontal cortex during a working memory task. We observed similar, albeit smaller, negative associations between insecticide metabolite concentrations and cortical activity related to attention/response inhibition and cognitive flexibility. Associations of fungicide and herbicide pesticides with cortical activation were essentially null.

Previous studies demonstrating associations between pesticide exposure and adverse neurobehavioral outcomes have relied primarily on neuropsychological assessments. Furthermore, most of the epidemiologic literature on pesticides and neurobehavioral outcomes among farmworkers occupationally exposed to pesticides has focused on OP insecticides. These studies have reported associations of OP pesticides with poorer working memory, processing speed, and attention problems (Corral et al., 2017; Meyer-Baron et al., 2015; Muñoz-Quezada et al., 2016; Wesseling et al., 2006). Our neuroimaging results are consistent with these previous studies, as each of the tasks we included are supported by neural functions that are mediated by the prefrontal cortex. Our findings indicating reduced cortical activation in relation to chlorpyrifos exposure during these tasks provide neurobiologic support for previous associations of OP pesticides with neuropsychological tests of attention and executive function (Fortenberry et al., 2014; Marks et al., 2010; Rauh et al., 2006; Sagiv et al., 2021). Moreover, our findings of reduced cortical activation in relation to pyrethroid insecticide exposure support further investigation of the impact of these pesticides on attention and working memory, for which there is currently a dearth of evidence among farmworkers or the general population (including those living in areas where long-lasting insecticide treated bednets are frequently used) (Eskenazi et al., 2018; Gunier et al., 2017; Lee et al., 2022, 2020; Lucero and Muñoz-Quezada, 2021).

Our study further demonstrates the utility of fNIRS as an optimal functional brain imaging method for use in resource-limited field settings common in epidemiologic studies (Baker et al., 2017). Compared with fMRI, which must be conducted in a clinic or hospital setting, fNIRS offers considerable advantages for testing participants who may be hard to reach, or who may be otherwise ill suited for fMRI due to restrictions on cost or time. In this study, we incorporated our fNIRS data collection into an existing field study, including traveling to each farm location. This mobile data collection approach enhances participation and affords collection of valuable functional neuroimaging data at a fraction of the cost of fMRI studies. As such, there is great potential for the application of fNIRS in resource-poor settings in tropical low- and middle-income countries, where pesticide use is increasing more rapidly than in any other area of the world (Food and Agriculture Organization (FAO), 2019).

To our knowledge, only two published studies of farmworkers have employed functional neuroimaging to examine associations of pesticide exposure and altered brain activity. In a study in North Carolina, investigators used rs-fMRI to examine brain network connectivity patterns among 48 Latino tobacco farmworkers occupationally exposed to pesticides and nicotine and 26 non-farmworkers (Bahrami et al., 2017). Researchers found evidence of more clustered and modular brain networks among farmworkers, suggesting more segregated neural processing and less sharing of information between brain regions (Bahrami et al., 2017). They also observed that acetylcholinesterase activity, a biomarker of exposure to OP pesticides and carbamates, was associated with differences in brain network community structure. In a previous analysis of 48 farmworkers from the PESTROP study, we observed largely null associations of hair and toenail concentrations of manganese, a biomarker of exposure to mancozeb (a bisdithiocarbamate fungicide widely used in Costa Rican agriculture) but also naturally occurring in water and food, and cortical activation during the same tasks described in the current analysis (Palzes et al., 2019). In addition to these studies of occupationally exposed populations, a few studies of children and adolescents environmentally exposed to pesticides have used functional neuroimaging to examine the effects of these chemicals on brain function. A study of 95 French children found that higher prenatal concentrations of urinary dialkylphosphate (DAP) metabolites, non-specific biomarkers of OP pesticides, were associated with reduced brain activation during an fMRI Go/No-Go task conducted at age 10–12 years (Binter et al., 2020). A study of 95 adolescents in California’s agricultural Salinas Valley, which used the same fNIRS technology as the current study, reported associations of residential proximity to OP applications during pregnancy with altered brain activation during tasks of executive function at age 15–17 years (Sagiv et al., 2019). Like ours, this study found reduced activation in the prefrontal cortex during a test of cognitive flexibility. An additional analysis of the 95 adolescents in California observed that prenatal and childhood exposure to dichloro-diphenyl-trichloroethane (DDT), an organochlorine pesticide, was associated with altered patterns of cortical activation during tasks of language comprehension and executive function (Binter et al., 2022). Lastly, a recent study of 48 farmworker and 30 non-farmworker children in North Carolina found differences in brain network connectivity and topology (assessed via rs-fMRI) between the two groups (Bahrami et al., 2022).

In our study of farmworkers, we did not detect associations for any of the measured pesticides and behavioral performance (e.g., accuracy or response time) on any of the three tasks we administered. It should be noted, however, that these tasks were not designed to test performance but rather to elicit a neural response during a challenge condition in relation to a control condition. This could explain the null associations that we found, compared to studies of standardized neurobehavioral tests. We were able to detect alterations in cortical activation in relation to some of these pesticides which suggests that changes in brain activity may be more sensitive to exposure than neurobehavioral tasks, where compensation by other areas of the brain could mask any apparent associations.

We primarily found reduced cortical activation in relation to OP and pyrethroid insecticide exposure. While we hypothesized that there would be altered cortical activation in relation to exposure in relevant brain regions, we did not specify a priori the direction of these alterations. Indeed, too few studies of this kind have been reported to hypothesize a priori the direction of these associations based on existing empirical data. Reduced activation could indicate that insecticide exposure reduced the ability of a brain region or network to marshal a typical neural response to a task demand. We hypothesize that the neurobiological insult that results from chronic pesticide exposure may be similar to that which results from neurogenetic conditions such as Fragile X and Turner syndrome; studies have documented reduced cortical activation among individuals with these conditions relative to their neurotypical counterparts (Haberecht et al., 2001; Kwon et al., 2001).

The primary limitation of our study is its small sample size. While by no means a small sample size for a neuroimaging study, it is a small size for detecting subtle associations of pesticide exposure with cortical brain activity. Despite this small sample size, we did observe suggestive associations for insecticides metabolites (i.e., TCPy, 3-PBA, and DDCA) after correcting for multiple comparisons, although confidence intervals were wide. Our small sample size also prevented us from assessing the joint effects of exposure to mixtures of pesticides. Future research should include large sample sizes to estimate associations of pesticide exposure with neural activity with more precision. Two other limitations of our study include the use of a convenience sampling scheme based on the availability of fNIRS equipment and technical staff and the PESTROP study recruitment of farmworkers at their workplace, both of which could have introduced selection bias such as the healthy worker effect. Notably, we did not find meaningful differences in farmworker characteristics between the subsets of participants who completed the fNIRS assessment (*n* = 48) and those who did not (*n* = 252).

It is important to note that the pesticides that we examined in our study metabolize rapidly in the body and the biomarkers analyzed reflect very recent exposures (Barr, 2008; Gillezeau et al., 2020). We attempted to reduce exposure measurement error and minimize intra-individual variability by collecting two spot urine samples, which has been shown to be a better predictor of long-term pesticide exposure than a single spot sample (Bradman et al., 2013; Meeker et al., 2005; Perrier et al., 2016). However, it is likely that some exposure misclassification remained, attenuating associations with cortical activation.

Lastly, there are some limitations of fNIRS compared with technologies such as fMRI. Most notably, fNIRS measures hemodynamic changes at the cortical surface, and thus does not capture changes in subcortical, deep-brain regions. If pesticides exert their effects in these subcortical regions, associations with neural activation could have been missed in this study. This limitation is balanced by the advantages of fNIRS; its lower cost and portability made this study considerably more feasible in this agricultural setting (Baker et al., 2017).

## 5. Conclusion

In our study of farmworkers, we observed that OP and pyrethroid insecticide exposure was associated with reduced cortical brain activation in the prefrontal cortex, which could underlie previously reported associations with cognitive and behavioral function. Given our small sample size, further exploration of the association between pesticides and brain activity, a potentially more sensitive endpoint than the more traditional neurobehavioral tests, is warranted. It is particularly important to understand the long-term health impact of pesticide exposure among farmworkers in Costa Rica, as it is one of the countries with the highest levels of agricultural pesticide use in the world.

## Supplementary Material

Supplementary Material

## Figures and Tables

**Table 1.** Sociodemographic and occupational characteristics of the study population, Zarcero County, Costa Rica.

*Characteristic*	fNIRS participants *n* (%)	Non-participants *n* (%)

All farmworkers	48 (16%)	252 (84%)
Sex		
Male	46 (96)	228 (90)
Female	2 (4)	24 (9)
Country of birth		
Costa Rica	34 (71)	143 (57)
Nicaragua	14 (29)	109 (43)
Age (years)		
< 35	25 (52)	137 (54)
≥ 35	23 (48)	115 (46)
Education level		
≤ 6th grade	31 (65)	176 (70)
7–11th grade	17 (35)	76 (30)
Handedness		
Right	45 (94)	229 (91)
Left	2 (4)	17 (7)
Missing	1 (2)	6 (2)
Poverty status		
≤Poverty line	13 (27)	84 (33)
>Poverty line	32 (67)	151 (60)
Missing	3 (6)	17 (7)
Marital status		
Married or cohabitating	27 (56)	156 (62)
Single	21 (44)	96 (38)
Smoker at time of enrollment		
No	39 (81)	198 (79)
Yes	9 (19)	54 (21)
Consuming any alcohol at time of enrollment		
No	3 (6)	79 (31)
Yes	45 (94)	173 (69)
Type of farm		
Organic	26 (54)	22 (9)
Conventional	22 (46)	230 (91)
Time working in agriculture (years)		
< 20	24 (50)	141 (56)
≥ 20	24 (50)	111 (44)
Time handling pesticides (years)		
< 20	21 (44)	131 (52)
≥ 20	23 (48)	97 (39)
Missing	4 (8)	24 (9)
Any pesticide application during the last 12 months		
No	9 (19)	37 (15)
Yes	39 (81)	215 (85)
Any pesticide application during the last week		
No	15 (31)	74 (29)
Yes	33 (69)	178 (71)
Ever used a computer or played videogames		
No	17 (35)	117 (46)
Yes	27 (56)	80 (32)
Missing	4 (8)	55 (22)
Self-reported neurological disorders (i.e., epilepsy)		
No	47 (98)	252 (100)
Yes	1 (2)	0 (0)

*n*, number of participants.

**Table 2.** Distribution of pesticide biomarker (specific gravity-adjusted) concentrations (not imputed) measured in farmworkers’ urine samples collected at one or two time points, Zarcero County, Costa Rica (*n* = 48).

Urinary biomarkers^a^	LOD	% >LOD		$\sigma_{\text{btw}}^{2}$ ^b^	$\sigma_{\text{within}}^{2}$ ^b^	ICC^b^	Average of two measurements^c^					
		1st measurement^d^	2nd measurement^e^				GM (GSD)	Min	P25	P50	P75	Max

TCPy	0.05	100.0	100.0	2.28	0.51	0.82	8.56 (3.08)	1.32	3.48	8.59	17.16	101.65
3-PBA	0.03	100.0	100.0	1.10	0.73	0.60	1.50 (2.46)	0.30	0.84	1.41	2.08	16.19
DCCA	0.04	100.0	100.0	1.09	0.72	0.60	2.44 (2.46)	0.53	1.34	2.14	3.27	34.86
ETU	0.08	97.9	97.7	1.71	1.26	0.57	1.17 (3.00)	0.14	0.59	1.12	1.98	44.91
2,4-D	0.02	91.7	97.7	0.53	2.04	0.21	0.26 (2.77)	< 0.02	0.16	0.25	0.31	9.10
TEB-OH	0.10	83.3	90.9	1.33	2.20	0.38	0.67 (3.34)	< 0.10	0.30	0.56	1.15	45.17
PTU	0.10	85.4	84.1	1.82	1.12	0.62	0.37 (3.10)	< 0.10	0.14	0.31	0.85	5.67
GLY	0.20	68.8	72.7	0.97	0.91	0.52	0.40 (2.29)	< 0.20	0.26	0.39	0.66	4.43
CFCA	0.10	60.4	65.9	0.51	0.85	0.38	0.17 (2.02)	< 0.10	0.11	0.15	0.25	1.27
AMPA	0.20	54.2	75.0	0.39	0.42	0.48	0.28 (1.75)	< 0.20	< 0.20	0.30	0.39	0.66
4F3PBA	0.01	54.2	65.9	0.24	1.40	0.14	0.02 (2.01)	< 0.01	0.01	0.01	0.02	0.15
OH-P	0.06	47.9	47.7	7.10	8.46	0.46	0.23 (15.26)	< 0.06	< 0.06	0.12	0.62	559.32
OH-T	0.03	25.0	22.7	0.04	8.75	0.00	<0.03 (6.95)	< 0.03	< 0.03	< 0.03	0.04	0.63

*Abbreviations*: LOD, limit of detection; ICC, intraclass correlation coefficient; GM, geometric mean; GSD, geometric standard deviation; ETU, ethylenethiourea; PTU, propylenethiourea; TCPy, 3,5,6-trichloro-2-pyridinol; 3-PBA, 3-phenoxybenzoic acid; 4F3PBA, 4-fluoro-3-phenoxybenzoic acid; DCCA, 3-(2,2-dichlorovinyl)–2,2-dimethylcyclopropanecarboxylic acid; CFCA, chloro-3,3,3-trifluoro-1-propen-1-yl]–2,2-dimethylcyclopropanecarboxylic acid; 2,4-D, 2,4-dichlorophenoxyacetic acid; OH-T, 5-hydroxy-thiabendazole; OH-P, 3-hydroxy-pyrimetanil; TEB-OH, hydroxy-tebuconazole; GLY, glyphosate; AMPA, aminomethylphosphonic acid.

a Units are ng/mL for all urinary pesticide biomarkers.

b Variances between- and within-worker and ICC were calculated and reported for log2-transformed specific gravity-adjusted urinary pesticide biomarkers concentrations (non-averaged).

c In the farmworkers for whom only one measurement was available, the single measurement was used in lieu of the average.

d Urine samples collected from 48 farmworkers.

e Urine samples collected from 44 farmworkers.

**Table 3.** Adjusted associations [β (95% CI)] for a two-fold increase in urinary pesticide biomarker (specific gravity-adjusted) concentrations with fNIRS brain activation (HbO) by task and region of interest in farmworkers from the Zarcero County, Costa Rica.

Contrast	Hemisphere	Position	*Urinary pesticide biomarkers*							
			Insecticides			Fungicides			Herbicides	
			TCPy	3-PBA	DCCA	ETU	PTU	TEB-OH	2,4-D	GLY

Encoding vs. recall (Sternberg test) (*n* = 48)	L	Inferior frontal pole	−1.89 (−3.65, −0.12)*	−2.06 (−4.24, 0.13)	−1.75 (−3.98, 0.47)	−0.65 (−2.48, 1.19)	0.19 (−1.57, 1.94)	−1.65 (−3.27, −0.04)*	−0.79 (−2.98, 1.40)	0.01 (−2.57, 2.60)
		Superior frontal pole	−1.66 (−3.15, −0.16)*	−2.31 (−4.11, −0.51)*†	−2.24 (−4.06, −0.42)*	−0.56 (−2.12, 1.01)	−0.04 (−1.53, 1.46)	−1.01 (−2.42, 0.40)	−1.31 (−3.14, 0.52)	1.01 (−1.17, 3.19)
		Broca/Broadmann	−1.21 (−2.87, 0.45)	−1.82 (−3.83, 0.18)	−1.38 (−3.43, 0.67)	−0.13 (−1.82, 1.57)	0.81 (−0.78, 2.40)	−1.09 (−2.61, 0.42)	−0.66 (−2.67, 1.34)	0.01 (−2.36, 2.38)
		Dorsolateral prefrontal	−2.30 (−3.90, −0.70)*†	−3.09 (−5.01, −1.17)*†	−3.03 (−4.98, −1.09)*†	−1.11 (−2.82, 0.60)	−0.29 (−1.95, 1.36)	−1.53 (−3.06, 0.00)*	−1.33 (−3.36, 0.71)	0.34 (−2.10, 2.77)
	R	Inferior frontal pole	−0.77 (−2.69, 1.14)	−2.40 (−4.65, −0.16)*	−2.22 (−4.50, 0.06)	−0.30 (−2.23, 1.62)	0.73 (−1.08, 2.55)	−1.97 (−3.63, −0.30)*	0.04 (−2.25, 2.33)	−0.16 (−2.85, 2.53)
		Superior frontal pole	−1.05 (−2.61, 0.51)	−1.91 (−3.78, −0.05)*	−1.98 (−3.85, −0.11)*	−0.11 (−1.70, 1.48)	0.29 (−1.22, 1.80)	−1.22 (−2.63, 0.19)	−0.72 (−2.60, 1.15)	−0.06 (−2.28, 2.17)
		Broca/Broadmann	−0.74 (−2.41, 0.93)	−2.28 (−4.22, −0.33)*	−2.12 (−4.09, −0.15)*	−0.24 (−1.92, 1.44)	0.24 (−1.35, 1.83)	−1.31 (−2.79, 0.18)	0.02 (−1.98, 2.02)	−0.46 (−2.81, 1.88)
		Dorsolateral prefrontal	−1.16 (−2.99, 0.67)	−2.33 (−4.49, −0.16)*	−2.03 (−4.24, 0.18)	−0.65 (−2.50, 1.20)	0.45 (−1.31, 2.21)	−1.40 (−3.05, 0.24)	−0.54 (−2.74, 1.67)	0.19 (−2.40, 2.78)
No-Go vs. Go (*n* = 48)	L	Inferior frontal pole	−0.24 (−2.24, 1.77)	−1.51 (−3.93, 0.90)	−1.12 (−3.57, 1.33)	0.76 (−1.23, 2.75)	0.17 (−1.73, 2.07)	−0.10 (−1.94, 1.73)	0.20 (−2.19, 2.58)	0.89 (−1.90, 3.67)
		Superior frontal pole	−0.99 (−3.36, 1.38)	−2.51 (−5.33, 0.32)	−2.43 (−5.28, 0.42)	−0.32 (−2.71, 2.06)	−1.48 (−3.70, 0.74)	−0.64 (−2.82, 1.53)	0.02 (−2.81, 2.86)	1.12 (−2.19, 4.44)
		Broca/Broadmann	−0.52 (−2.68, 1.64)	−1.76 (−4.36, 0.84)	−1.60 (−4.23, 1.02)	0.43 (−1.73, 2.59)	−0.06 (−2.11, 1.99)	−0.94 (−2.90, 1.02)	0.56 (−2.01, 3.13)	0.72 (−2.29, 3.73)
		Dorsolateral prefrontal	−0.98 (−3.16, 1.20)	−1.98 (−4.61, 0.65)	−1.64 (−4.31, 1.02)	0.43 (−1.77, 2.62)	−0.60 (−2.68, 1.48)	0.30 (−1.71, 2.31)	−0.74 (−3.35, 1.86)	0.72 (−2.34, 3.78)
	R	Inferior frontal pole	−0.10 (−1.69, 1.48)	0.25 (−1.69, 2.19)	0.12 (−1.83, 2.07)	0.38 (−1.20, 1.95)	0.25 (−1.25, 1.75)	0.29 (−1.16, 1.74)	−0.55 (−2.42, 1.32)	1.52 (−0.64, 3.68)
		Superior frontal pole	−0.57 (−2.46, 1.32)	−0.71 (−3.03, 1.60)	−0.62 (−2.95, 1.71)	0.09 (−1.81, 1.98)	−0.92 (−2.70, 0.86)	−0.12 (−1.86, 1.61)	−0.54 (−2.79, 1.71)	−0.05 (−2.69, 2.60)
		Broca/Broadmann	−0.97 (−2.83, 0.88)	−0.66 (−2.95, 1.63)	−0.81 (−3.12, 1.49)	−0.26 (−2.14, 1.61)	−0.53 (−2.30, 1.24)	−0.13 (−1.85, 1.58)	−0.59 (−2.81, 1.63)	1.27 (−1.32, 3.86)
		Dorsolateral prefrontal	−1.60 (−3.43, 0.22)	−0.81 (−3.11, 1.49)	−0.96 (−3.27, 1.35)	−1.47 (−3.30, 0.36)	−1.85 (−3.54, −0.15)*	−0.83 (−2.54, 0.87)	−1.31 (−3.52, 0.89)	−0.92 (−3.53, 1.70)
Matching vs. control (Wisconsin Card Sort test) (*n* = 41)	L	Inferior frontal pole	−1.83 (−4.89, 1.24)	−1.82 (−5.85, 2.21)	−2.93 (−6.96, 1.09)	−2.04 (−5.27, 1.18)	−1.64 (−4.92, 1.63)	−1.49 (−4.78, 1.79)	−0.08 (−3.69, 3.53)	−1.52 (−5.84, 2.80)
		Superior frontal pole	−1.41 (−4.05, 1.23)	−2.16 (−5.58, 1.26)	−2.53 (−5.98, 0.92)	−1.99 (−4.74, 0.76)	−0.83 (−3.67, 2.00)	0.08 (−2.77, 2.93)	0.05 (−3.04, 3.15)	−1.68 (−5.36, 2.01)
		Broca/Broadmann	−0.75 (−3.81, 2.31)	−1.29 (−5.27, 2.69)	−2.12 (−6.13, 1.88)	−2.94 (−6.02, 0.15)	−2.83 (−5.95, 0.29)	−1.30 (−4.53, 1.94)	0.48 (−3.06, 4.03)	−2.31 (−6.52, 1.89)
		Dorsolateral prefrontal	−0.36 (−2.99, 2.26)	−2.44 (−5.77, 0.89)	−2.52 (−5.90, 0.86)	−2.33 (−4.99, 0.33)	−1.56 (−4.31, 1.18)	−0.35 (−3.14, 2.44)	−0.23 (−3.27, 2.80)	−1.81 (−5.41, 1.80)
	R	Inferior frontal pole	−1.41 (−4.32, 1.50)	−1.45 (−5.27, 2.38)	−2.34 (−6.18, 1.49)	−1.37 (−4.46, 1.71)	−1.43 (−4.53, 1.67)	−0.95 (−4.07, 2.18)	0.04 (−3.37, 3.46)	−0.53 (−4.63, 3.58)
		Superior frontal pole	−0.60 (−3.29, 2.10)	−1.11 (−4.62, 2.39)	−1.52 (−5.06, 2.03)	0.16 (−2.69, 3.01)	−1.24 (−4.08, 1.60)	0.59 (−2.28, 3.45)	0.38 (−2.73, 3.50)	0.43 (−3.33, 4.18)
		Broca/Broadmann	−0.06 (−2.86, 2.75)	−0.55 (−4.20, 3.11)	−1.49 (−5.18, 2.19)	−1.11 (−4.05, 1.83)	−0.39 (−3.36, 2.59)	−0.43 (−3.41, 2.55)	0.35 (−2.89, 3.59)	0.64 (−3.25, 4.54)
		Dorsolateral prefrontal	−0.81 (−3.29, 1.66)	−0.64 (−3.88, 2.60)	−1.39 (−4.66, 1.87)	−0.73 (−3.35, 1.89)	−1.79 (−4.36, 0.79)	0.15 (−2.50, 2.79)	1.25 (−1.59, 4.10)	−1.18 (−4.62, 2.26)

*Abbreviations*: fNIRS, functional Near-Infrared Spectroscopy; *n*, number of participants; L, left; R, right; ETU, ethylenethiourea; PTU, propylenethiourea; TCPy, 3,5,6- trichloro-2-pyridinol; 3-PBA, 3-phenoxybenzoic acid; DCCA, 3-(2,2-dichlorovinyl)–2,2-dimethylcyclopropanecarboxylic acid; 2,4-D, 2,4-dichlorophenoxyacetic acid; TEB-OH, hydroxy-tebuconazole; GLY, glyphosate.

Models adjusted for age (continuous variable) and education level (≤6th grade, 7–11th grade).

* non-FDR corrected p < 0.05

† FDR-corrected p < 0.05.
