# Supplementary material for: Pesticide exposure and cortical brain activation among farmworkers in Costa Rica
Source: Neurotoxicology. Author manuscript; Available in PMC 2023 Mar 15. (PMC10014323; doi:10.1016/j.neuro.2022.10.004)
Supplement: Supplementary Material [file NIHMS1876148-supplement-Supplementary_Material.pdf]

SUPPLEMENTARY MATERIAL

Pesticide exposure and cortical brain activation among farmworkers in Costa Rica

Ana M. Mora,[a†] Joseph M. Baker,[b†] Carly Hyland,[c] María G. Rodríguez-Zamora,[d] Daniel Rojas-Valverde,[e] Mirko S. Winkler,[f,g] Philipp Staudacher,[h] Vanessa A. Palzes,[i] Randall Gutiérrez-Vargas,[e] Christian Lindh,[j] Allan L. Reiss,[b,k] Brenda Eskenazi,[a] Samuel Fuhrimann,[f, g] Sharon K. Sagiv[a]

† = Co-first authorship

[a]Center for Environmental Research and Community Health (CERCH), School of Public Health, University of California, Berkeley, 1995 University Avenue, Suite 265, Berkeley, CA 94720, USA

[b]Center for Interdisciplinary Brain Sciences Research, Division of Brain Sciences, Department of Psychiatry and Behavioral Sciences, School of Medicine, Stanford University, 401 Quarry Road, Stanford, CA 94305, USA

[c]School of Public Health and Population Science, Boise State University, 1910 W University Dr, Boise, ID 83725, USA

[d]Escuela de Ingeniería en Seguridad Laboral e Higiene Ambiental (EISLHA), Instituto Tecnológico de Costa Rica, Calle 15, Avenida 14, 1 km Sur de la Basílica de los Ángeles, Provincia de Cartago, Cartago 30101, Costa Rica

[e]Centro de Investigación y Diagnóstico en Salud y Deporte, Escuela Ciencias del Movimiento Humano y Calidad de Vida, Campus Benjamin Nuñez, Universidad Nacional, Heredia 86-3000, Costa Rica

[f]Department of Epidemiology and Public Health, Swiss Tropical and Public Health Institute, Socinstrasse 55, 4051 Basel, Switzerland

[g]University of Basel, Peterspl. 1, 4001 Basel, Switzerland

[h]Swiss Federal Institute of Aquatic Science and Technology (EAWAG), Ueberlandstrasse 133, 8600 Dübendorf, Switzerland

[i]Drug and Alcohol Research Team at the Kaiser Permanente Northern California's Division of Research, 2000 Broadway, Oakland, CA 94612, USA

[j]Division of Occupational and Environmental Medicine, Institute of Laboratory Medicine, Lund University, Scheelevägen 2, 22363 Lund, Sweden

[k]Department of Radiology, School of Medicine, Stanford University, 401 Quarry Road, Stanford, CA 94305, USA

Table S1. Description of pesticides and urinary biomarkers assessed in this study.

Group	Use	Chemical family	Pesticide	Urinary biomarker
Insecticides	Carrots, potatoes, cilantro, lettuce, onions	Organophosphates	Chlorpyrifos	3,5,6-trichloro-2-pyridinol (TCPy)
	Carrots, potatoes, beets, lettuce, cilantro, celery, pastures	Synthetic pyrethroids	Permethrin, cypermethrin, deltamethrin, allethrin, resmethrin, fenvalerate	3-phenoxybenzoic acid (3-PBA)
			Permethrin, cypermethrin, cyfluthrin	3-(2,2-dichlorovinyl)-2,2-dimethylcyclopropanecarboxylic acid (DCCA)
			Bifenthrin	Chloro-3,3,3-trifluoro-1-propene-1-yl-2,2-dimethyl cyclopropane carboxylic acid (CFCA)
			Cyfluthrin	4-fluoro-3-phenoxybenzoic acid (4F3PBA)
Fungicides	Potatoes, carrots, beets, lettuce, cilantro, onions	Dithiocarbamates	Mancozeb, maneb	Ethylenethiourea (ETU)
			Propineb	Propylenethiourea (PTU)
		Anilinopyrimidines	Pyrimethanil	Hydroxypyrimethanil (OH-P)
	Onions	Benzimidazoles	Thiabendazole	5-hydroxythiabendazole (OH-T)
	Potatoes, lettuce	Triazole	Tebuconazole	Hydroxy-tebuconazole (TEB-OH)
Herbicides	Potatoes, pastures	Dichlorophenoxyacetic acid	2,4-D	2,4-D (parent compound)
	Potatoes, carrots, cilantro, onions, pastures	(non accepted) Glycines	Glyphosate	Glyphosate (parent compound)
				Aminomethylphosphonic acid (AMPA)

Table S2. Distribution of pesticide biomarker (crude) concentrations (not imputed) measured in farmworkers' urine samples collected at one or two time points, Zarcero County, Costa Rica (*n* = 48).

**Urinary biomarkers**[a]	**Average of two measurements**[b]					
	**GM (GSD)**	**Min**	**P25**	**P50**	**P75**	**Max**
TCPy	8.23 (3.66)	0.43	3.42	8.86	16.26	152.19
3-PBA	1.43 (2.87)	0.14	0.69	1.53	2.53	13.27
DCCA	2.29 (2.81)	0.24	1.15	2.12	3.82	25.94
4F3PBA	0.02 (2.44)	<0.01	0.01	0.02	0.03	0.09
CFCA	0.17 (2.47)	<0.10	0.10	0.17	0.25	1.81
ETU	1.08 (3.18)	0.10	0.54	1.00	2.47	19.31
PTU	0.34 (2.98)	<0.10	0.14	0.35	0.70	6.67
OH-T	<0.03 (7.42)	<0.03	<0.03	<0.03	0.05	0.53
OH-P	0.21 (15.27)	<0.06	<0.06	0.15	0.59	658.08
TEB-OH	0.64 (3.70)	<0.10	0.26	0.61	1.42	17.42
2,4-D	0.25 (3.13)	<0.02	0.16	0.24	0.34	13.39
GLY	0.39 (2.77)	<0.20	0.23	0.36	0.76	6.85
AMPA	0.27 (2.14)	<0.20	<0.20	0.31	0.47	0.78

Abbreviations: GM, geometric mean; GSD, geometric standard deviation; ETU, ethylenethiourea; PTU, propylenethiourea; TCPy, 3,5,6-trichloro-2-pyridinol; 3-PBA, 3-phenoxybenzoic acid; 4F3PBA, 4-fluoro-3-phenoxybenzoic acid; DCCA, 3-(2,2-dichlorovinyl)-2,2-dimethylcyclopropanecarboxylic acid; CFCA, chloro-3,3,3-trifluoro-1-propen-1-yl]-2,2-dimethylcyclopropanecarboxylic acid; 2,4-D, 2,4-dichlorophenoxyacetic acid; OH-T, 5-hydroxy-thiabendazole; OH-P, 3-hydroxy-pyrimetanil; TEB-OH, hydroxy-tebuconazole; GLY, glyphosate; AMPA, aminomethylphosphonic acid.

[a]Units are ng/mL for all urinary pesticide biomarkers.

[b]In the farmworkers for whom only one measurement was available, the single measurement was used in lieu of the average.

Table S3. Spearman correlations for pesticide biomarker (specific gravity-adjusted) concentrations (not imputed) measured in farmworkers' urine samples collected at two time points, Zarcero County, Costa Rica (*n* = 44).[a]

Urinary biomarkers	Rho	p-value
TCPy	0.81	<0.01
3-PBA	0.56	<0.01
DCCA	0.51	<0.01
ETU	0.48	<0.01
2,4-D	-0.12	0.44
TEB-OH	0.17	0.28
PTU	0.61	<0.01
GLY	0.46	<0.01
CFCA	0.34	0.02
AMPA	0.36	0.02
4F3PBA	0.18	0.25
OH-P	0.33	0.03
OH-T	0.25	0.10

Abbreviations: ETU, ethylenethiourea; PTU, propylenethiourea; TCPy, 3,5,6-trichloro-2-pyridinol; 3-PBA, 3-phenoxybenzoic acid; 4F3PBA, 4-fluoro-3-phenoxybenzoic acid; DCCA, 3-(2,2-dichlorovinyl)-2,2-dimethylcyclopropanecarboxylic acid; CFCA, chloro-3,3,3-trifluoro-1-propen-1-yl]-2,2-dimethylcyclopropanecarboxylic acid; 2,4-D, 2,4-dichlorophenoxyacetic acid; OH-T, 5-hydroxy-thiabendazole; OH-P, 3-hydroxy-pyrimetanil; TEB-OH, hydroxy-tebuconazole; GLY, glyphosate; AMPA, aminomethylphosphonic acid.

[a]Four farmworkers only provided one urine sample and were excluded from this correlation analysis.

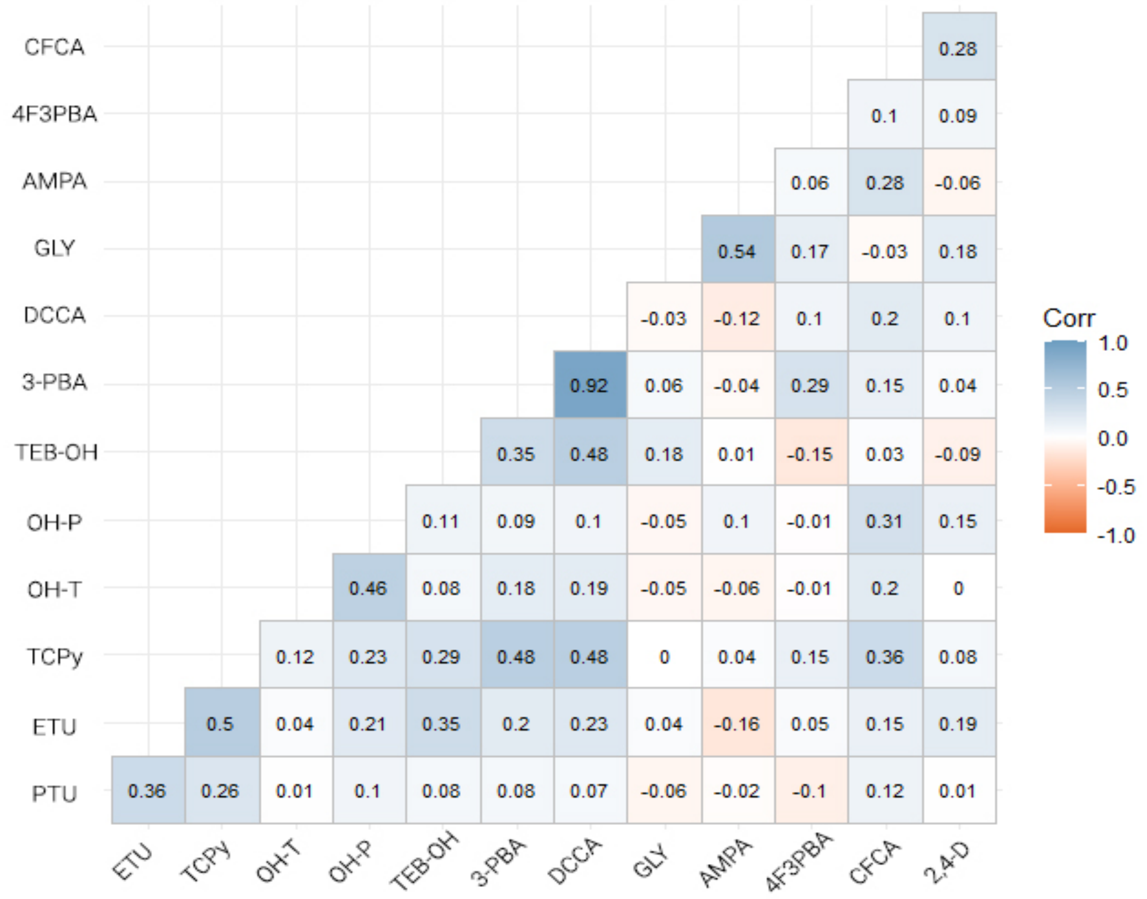

Figure S1. Spearman correlation coefficients for averaged urinary pesticide biomarker (specific gravity-adjusted) concentrations (not imputed) in farmworkers from the Zarcero County, Costa Rica (*n* = 48). *Abbreviations*: ETU, ethylenethiourea; PTU, propylenethiourea; TCPy, 3,5,6-trichloro-2-pyridinol;3-PBA, 3-phenoxybenzoic acid; 4F3PBA, 4-fluoro-3-phenoxybenzoic acid; DCCA, 3-(2,2-dichlorovinyl)-2,2-dimethylcyclopropanecarboxylic acid; CFCA, chloro-3,3,3-trifluoro-1-propen-1-yl]-2,2-dimethylcyclopropanecarboxylic acid; 2,4-D, 2,4-dichlorophenoxyacetic acid; OH-T, 5-hydroxy-thiabendazole; OH-P, 3-hydroxy-pyrimetanil; TEB-OH, hydroxy-tebuconazole; GLY, glyphosate; AMPA, aminomethylphosphonic acid.

Table S4. Distribution [GM (GSD)] of pesticide biomarker (specific gravity-adjusted) concentrations (imputed) measured in workers' urine samples by sociodemographic and occupational characteristics, Zarcero County, Costa Rica (n = 48).

Characteristic	***n***	***Urinary pesticide biomarkers***															
		**Insecticides**						**Fungicides**						**Herbicides**			
		**TCPy**	***p***	**3-PBA**	***p***	**DCCA**	***p***	**ETU**	***p***	**PTU**	***p***	**TEB-OH**	***p***	**2,4-D**	***p***	**GLY**	***p***
Sex																	
Male	46	8.78 (3.13)	0.54	1.54 (2.49)	0.16	2.53 (2.46)	0.09	1.17 (3.08)	0.82	0.36 (3.26)	0.67	0.69 (3.38)	0.21	0.28 (2.56)	0.28	0.41 (2.17)	0.11
Female	2	4.81 (1.16)		0.78 (1.07)		1.02 (1.30)		1.00 (1.17)		0.42 (1.22)		0.30 (1.20)		0.16 (1.04)		0.96 (1.81)	
Country of birth																	
Costa Rica	34	8.77 (3.05)	0.87	1.48 (2.36)	0.66	2.41 (2.36)	0.90	1.25 (2.97)	0.48	0.33 (2.63)	0.65	0.64 (3.20)	0.60	0.30 (2.58)	0.22	0.47 (2.36)	0.12
Nicaragua	14	8.09 (3.26)		1.54 (2.81)		2.51 (2.81)		0.97 (3.16)		0.44 (4.71)		0.75 (3.80)		0.22 (2.35)		0.32 (1.65)	
Age (years)																	
<35	25	8.08 (2.57)	0.92	1.28 (2.47)	0.08	2.03 (2.47)	0.08	1.13 (2.50)	0.71	0.34 (3.49)	0.64	0.63 (3.11)	0.67	0.25 (2.69)	0.24	0.41 (1.82)	0.87
≥35	23	9.12 (3.71)		1.78 (2.43)		2.97 (2.40)		1.20 (3.64)		0.38 (2.92)		0.71 (3.65)		0.30 (2.36)		0.44 (2.61)	
Education level																	
≤6th grade	31	10.36 (3.10)	0.13	1.66 (2.31)	0.22	2.74 (2.18)	0.09	1.23 (3.20)	0.61	0.40 (2.81)	0.36	0.72 (3.78)	0.47	0.27 (2.29)	0.18	0.42 (2.49)	0.62
7-11th grade	17	6.06 (2.86)		1.23 (2.73)		1.96 (2.95)		1.05 (2.73)		0.30 (3.95)		0.58 (2.57)		0.28 (3.01)		0.43 (1.64)	
Poverty status[a]																	
≤Poverty line	15	13.58 (3.34)	0.05	1.71 (2.96)	0.90	2.87 (2.98)	0.69	2.01 (3.68)	0.02	0.61 (3.29)	0.03	0.97 (4.30)	0.25	0.29 (3.23)	0.81	0.51 (2.25)	0.22
>Poverty line	33	6.94 (2.82)		1.41 (2.26)		2.26 (2.24)		0.91 (2.49)		0.28 (2.93)		0.56 (2.85)		0.27 (2.24)		0.39 (2.16)	
Marital status																	
Married	27	11.27 (3.37)	0.07	1.80 (2.40)	0.04	3.06 (2.33)	0.01	1.27 (3.57)	0.73	0.44 (3.06)	0.15	0.77 (3.81)	0.29	0.31 (3.01)	0.34	0.39 (2.52)	0.19
Single	21	6.01 (2.47)		1.18 (2.45)		1.82 (2.48)		1.04 (2.34)		0.28 (3.27)		0.56 (2.73)		0.23 (1.86)		0.47 (1.76)	
Type of farm																	
Organic	26	4.77 (2.28)	<0.01	1.01 (2.12)	<0.01	1.66 (1.98)	<0.01	0.79 (2.18)	0.01	0.27 (2.72)	0.08	0.57 (2.41)	0.45	0.23 (1.74)	0.25	0.48 (1.75)	0.05
Conventional	22	17.09 (2.85)		2.39 (2.35)		3.83 (2.54)		1.83 (3.53)		0.50 (3.51)		0.81 (4.47)		0.33 (3.36)		0.36 (2.66)	
Time working in agriculture (years)																	
<20	24	7.96 (2.62)	0.83	1.25 (2.50)	0.05	1.99 (2.50)	0.05	1.17 (2.50)	0.53	0.36 (3.48)	0.96	0.65 (3.17)	0.78	0.26 (2.73)	0.44	0.41 (1.83)	0.99
≥20	24	9.21 (3.60)		1.79 (2.38)		2.98 (2.36)		1.16 (3.59)		0.36 (2.97)		0.69 (3.57)		0.29 (2.35)		0.43 (2.57)	
Time handling pesticides (years)[b]																	
<20	25	8.08 (2.57)	0.92	1.28 (2.47)	0.08	2.03 (2.47)	0.08	1.13 (2.50)	0.71	0.34 (3.49)	0.64	0.63 (3.11)	0.67	0.25 (2.69)	0.24	0.41 (1.82)	0.87
≥20	23	9.12 (3.71)		1.78 (2.43)		2.97 (2.40)		1.20 (3.64)		0.38 (2.92)		0.71 (3.65)		0.30 (2.36)		0.44 (2.61)	

Any pesticide application at work during the last 12 months																	
No	9	5.58 (1.70)	0.27	0.89 (1.71)	0.03	1.55 (1.79)	0.11	0.67 (1.80)	0.07	0.25 (2.23)	0.33	0.34 (1.66)	0.04	0.26 (1.98)	0.90	0.44 (2.27)	0.57
Yes	39	9.45 (3.33)		1.69 (2.54)		2.71 (2.55)		1.32 (3.18)		0.39 (3.38)		0.78 (3.54)		0.28 (2.66)		0.42 (2.20)	
Any pesticide application at work during the last week																	
No	15	5.57 (2.02)	0.07	1.01 (2.48)	0.01	1.75 (2.24)	0.05	0.77 (1.92)	0.09	0.30 (2.08)	0.63	0.44 (2.13)	0.09	0.25 (1.75)	0.76	0.39 (2.18)	0.98
Yes	33	10.41 (3.42)		1.79 (2.34)		2.83 (2.49)		1.40 (3.37)		0.39 (3.70)		0.81 (3.75)		0.28 (2.87)		0.43 (2.22)	

Abbreviations: GM, geometric mean; GSD, geometric standard deviation; *n*, number of participants; ETU, ethylenethiourea; PTU, propylenethiourea; TCPy, 3,5,6-trichloro-2-pyridinol; 3-PBA, 3-phenoxybenzoic acid; DCCA, 3-(2,2-dichlorovinyl)-2,2-dimethylcyclopropanecarboxylic acid; 2,4-D, 2,4-dichlorophenoxyacetic acid; TEB-OH, hydroxy-tebuconazole; GLY, glyphosate.

Median comparisons using the Wilcoxon Mann-Whitney test. Units are ng/mL for all urinary pesticide biomarkers.

[a]Imputed data for missing information from 3 participants.

[b]Imputed data for missing information from 4 participants.

Table S5. Adjusted associations [β (95% CrI)] for a two-fold increase in urinary pesticide biomarker (specific gravity-adjusted) concentrations with fNIRS brain activation (HbO) by task and region of interest in farmworkers from the Zarcero County, Costa Rica, assessed using Bayesian Hierarchical Modeling.

Contrast	Hemisphere	Position	*Urinary pesticide biomarkers*							
			Insecticides			Fungicides			Herbicides	
			TCPy	3-PBA	DCCA	ETU	PTU	TEB-OH	2,4-D	GLY
Encoding vs. recall (Sternberg test) (*n* = 48)	L	Inferior frontal pole	-1.46 (-3.21, 0.30)	-0.83 (-3.14, 1.48)	-0.12 (-2.51, 2.26)	0.77 (-1.09, 2.64)	0.55 (-0.99, 2.10)	-1.15 (-2.93, 0.63)	-0.86 (-2.82, 1.10)	0.35 (-1.75, 2.45)
		Superior frontal pole	-1.28 (-2.79, 0.23)	-1.14 (-3.31, 1.02)	-0.82 (-3.07, 1.43)	0.90 (-0.74, 2.53)	0.40 (-0.91, 1.71)	-0.48 (-2.02, 1.07)	-1.32 (-3.00, 0.35)	1.17 (-0.62, 2.95)
		Broca/Broadmann	-1.13 (-2.79, 0.53)	-1.14 (-3.39, 1.12)	-0.09 (-2.42, 2.24)	0.90 (-0.88, 2.67)	1.01 (-0.44, 2.46)	-0.73 (-2.42, 0.95)	-0.85 (-2.70, 0.99)	0.24 (-1.73, 2.21)
		Dorsolateral prefrontal	-1.57 (-3.18, 0.05)	-1.33 (-3.56, 0.90)	-1.09 (-3.4, 1.22)	0.71 (-1.03, 2.44)	0.35 (-1.06, 1.76)	-0.55 (-2.19, 1.10)	-1.20 (-2.99, 0.60)	0.69 (-1.23, 2.61)
	R	Inferior frontal pole	-0.46 (-2.31, 1.39)	-1.04 (-3.4, 1.32)	-0.57 (-3.00, 1.87)	0.71 (-1.24, 2.66)	0.79 (-0.84, 2.43)	-1.26 (-3.12, 0.61)	-0.41 (-2.48, 1.66)	0.11 (-2.11, 2.33)
		Superior frontal pole	-0.79 (-2.40, 0.82)	-0.76 (-2.99, 1.46)	-0.83 (-3.14, 1.47)	1.13 (-0.6, 2.86)	0.47 (-0.93, 1.87)	-0.85 (-2.49, 0.79)	-1.05 (-2.83, 0.74)	0.14 (-1.77, 2.04)
		Broca/Broadmann	-0.39 (-2.12, 1.33)	-1.14 (-3.43, 1.15)	-0.77 (-3.14, 1.60)	0.69 (-1.14, 2.52)	0.41 (-1.1, 1.92)	-0.60 (-2.35, 1.14)	-0.32 (-2.24, 1.60)	-0.18 (-2.23, 1.88)
		Dorsolateral prefrontal	-0.76 (-2.59, 1.08)	-1.15 (-3.51, 1.20)	-0.53 (-2.96, 1.89)	0.37 (-1.56, 2.31)	0.71 (-0.91, 2.33)	-0.64 (-2.49, 1.21)	-0.58 (-2.63, 1.48)	0.37 (-1.83, 2.57)
No-Go vs. Go (*n* = 48)	L	Inferior frontal pole	-0.50 (-2.50, 1.51)	-1.17 (-3.62, 1.28)	-0.69 (-3.21, 1.83)	1.00 (-1.08, 3.09)	0.26 (-1.52, 2.04)	0.33 (-1.67, 2.34)	0.06 (-2.20, 2.31)	0.65 (-1.77, 3.07)
		Superior frontal pole	-0.58 (-2.80, 1.65)	-1.19 (-3.78, 1.39)	-1.12 (-3.77, 1.53)	0.43 (-1.86, 2.71)	-0.80 (-2.81, 1.2)	0.21 (-2.0, 2.42)	0.25 (-2.30, 2.80)	0.94 (-1.79, 3.68)
		Broca/Broadmann	-0.54 (-2.66, 1.57)	-0.91 (-3.42, 1.61)	-0.69 (-3.28, 1.89)	0.86 (-1.32, 3.04)	0.08 (-1.81, 1.97)	-0.32 (-2.42, 1.79)	0.30 (-2.10, 2.70)	0.66 (-1.91, 3.24)
		Dorsolateral prefrontal	-0.95 (-3.05, 1.16)	-1.41 (-3.92, 1.10)	-1.14 (-3.72, 1.44)	1.07 (-1.10, 3.24)	-0.11 (-1.99, 1.78)	0.91 (-1.19, 3.01)	-0.47 (-2.86, 1.92)	0.44 (-2.13, 3.00)
	R	Inferior frontal pole	-0.39 (-2.09, 1.31)	0.07 (-2.21, 2.35)	-0.14 (-2.49, 2.22)	0.41 (-1.41, 2.22)	0.24 (-1.25, 1.73)	0.07 (-1.65, 1.80)	-0.44 (-2.34, 1.45)	1.14 (-0.88, 3.17)
		Superior frontal pole	-0.31 (-2.27, 1.65)	-0.36 (-2.78, 2.07)	-0.29 (-2.78, 2.21)	0.58 (-1.46, 2.63)	-0.67 (-2.40, 1.07)	-0.03 (-1.99, 1.93)	-0.49 (-2.69, 1.71)	-0.07 (-2.43, 2.29)
		Broca/Broadmann	-0.79 (-2.72, 1.14)	-0.24 (-2.64, 2.17)	-0.45 (-2.93, 2.03)	0.24 (-1.78, 2.25)	-0.21 (-1.92, 1.50)	0.13 (-1.80, 2.07)	-0.23 (-2.40, 1.94)	1.01 (-1.32, 3.33)
		Dorsolateral prefrontal	-0.31 (-2.27, 1.65)	-0.36 (-2.78, 2.07)	-0.29 (-2.78, 2.21)	0.58 (-1.46, 2.63)	-0.67 (-2.40, 1.07)	-0.03 (-1.99, 1.93)	-0.49 (-2.69, 1.71)	-0.07 (-2.43, 2.29)
Matching vs. control	L	Inferior frontal pole	0.54 (-1.73, 2.81)	0.08 (-2.54, 2.69)	-0.29 (-2.97, 2.39)	-0.57 (-2.89, 1.75)	1.11 (-0.95, 3.16)	-0.89 (-3.15, 1.36)	-1.64 (-4.25, 0.97)	-2.25 (-5.05, 0.55)

(Wisconsin Card Sort test) (*n* = 41)		Superior frontal pole	-0.47 (-2.57, 1.63)	0.54 (-1.97, 3.04)	-0.05 (-2.62, 2.53)	-1.93 (-4.10, 0.24)	1.72 (-0.16, 3.6)	0.92 (-1.17, 3.01)	-1.51 (-3.89, 0.87)	-2.09 (-4.65, 0.47)
		Broca/Broadmann	1.14 (-1.10, 3.38)	0.41 (-2.18, 3.00)	-0.07 (-2.73, 2.59)	-0.95 (-3.24, 1.34)	0.62 (-1.40, 2.63)	-1.19 (-3.41, 1.03)	0.36 (-2.20, 2.92)	-1.92 (-4.67, 0.83)
		Dorsolateral prefrontal	1.51 (-0.55, 3.58)	0.79 (-1.7, 3.28)	0.26 (-2.3, 2.81)	-2.04 (-4.19, 0.10)	0.35 (-1.50, 2.20)	0.34 (-1.73, 2.40)	-2.72 (-5.06, -0.37)*	-2.97 (-5.49, -0.46)*
	R	Inferior frontal pole	-0.51 (-2.72, 1.70)	-0.5 (-3.07, 2.08)	-0.99 (-3.63, 1.65)	1.23 (-1.04, 3.50)	1.76 (-0.23, 3.75)	0.59 (-1.6, 2.79)	-1.69 (-4.22, 0.84)	-2.28 (-4.99, 0.44)
		Superior frontal pole	0.58 (-1.61, 2.77)	-0.08 (-2.65, 2.48)	-0.28 (-2.91, 2.35)	0.21 (-2.04, 2.46)	1.54 (-0.43, 3.51)	0.85 (-1.33, 3.03)	-1.09 (-3.60, 1.41)	-3.46 (-6.15, -0.78)*
		Broca/Broadmann	0.59 (-1.67, 2.85)	1.12 (-1.49, 3.72)	0.84 (-1.83, 3.51)	-0.40 (-2.71, 1.91)	1.29 (-0.75, 3.32)	-0.57 (-2.81, 1.67)	-1.58 (-4.17, 1.01)	-1.10 (-3.87, 1.68)
		Dorsolateral prefrontal	-0.93 (-3.23, 1.38)	0.42 (-2.21, 3.05)	-0.31 (-3.01, 2.38)	0.41 (-1.93, 2.76)	0.66 (-1.42, 2.74)	0.74 (-1.54, 3.02)	-0.41 (-3.06, 2.24)	-1.61 (-4.45, 1.23)

Abbreviations: fNIRS, functional Near-Infrared Spectroscopy; *n*, number of participants; L, left; R, right; ETU, ethylenethiourea; PTU, propylenethiourea; TCPy, 3,5,6-trichloro-2-pyridinol; 3-PBA, 3-phenoxybenzoic acid; DCCA, 3-(2,2-dichlorovinyl)-2,2-dimethylcyclopropanecarboxylic acid; 2,4-D, 2,4-dichlorophenoxyacetic acid; TEB-OH, hydroxy-tebuconazole; GLY, glyphosate.
Models adjusted for age (continuous variable) and education level (≤6th grade, 7-11th grade).
*non-FDR corrected p<0.05.

Table S6. Adjusted associations [β (95% CI)] for a two-fold increase in urinary pesticide biomarker (specific gravity-adjusted) concentrations (imputed) with fNIRS brain activation (HbR) by task and region of interest in farmworkers from the Zarcero County, Costa Rica.

Contrast	Hemisphere	Position	*Urinary pesticide biomarkers*							
			**Insecticides**			**Fungicides**			**Herbicides**	
			**TCPy**	**3-PBA**	**DCCA**	**ETU**	**PTU**	**TEB-OH**	**2,4-D**	**GLY**
Encoding vs. recall (Sternberg test) (n = 48)	L	Inferior frontal pole	-0.64 (-2.21, 0.94)	-0.66 (-2.60, 1.27)	-0.49 (-2.44, 1.46)	-0.30 (-1.88, 1.28)	0.46 (-1.04, 1.96)	-0.39 (-1.84, 1.05)	0.90 (-0.97, 2.76)	0.17 (-2.04, 2.38)
		Superior frontal pole	0.48 (-1.11, 2.07)	-0.12 (-2.08, 1.83)	0.10 (-1.86, 2.07)	0.50 (-1.09, 2.08)	1.21 (-0.26, 2.67)	0.14 (-1.32, 1.60)	0.38 (-1.51, 2.28)	0.80 (-1.42, 3.01)
		Broca/Broadmann	-0.21 (-1.61, 1.19)	-0.33 (-2.04, 1.38)	-0.14 (-1.87, 1.58)	0.18 (-1.22, 1.58)	0.24 (-1.08, 1.57)	-0.21 (-1.49, 1.07)	0.86 (-0.78, 2.51)	-0.09 (-2.04, 1.86)
		Dorsolateral prefrontal	0.43 (-0.83, 1.69)	1.04 (-0.48, 2.56)	1.05 (-0.48, 2.58)	0.31 (-0.95, 1.57)	0.65 (-0.53, 1.84)	0.82 (-0.31, 1.96)	-0.85 (-2.34, 0.63)	-0.97 (-2.71, 0.77)
	R	Inferior frontal pole	0.36 (-0.85, 1.57)	0.86 (-0.60, 2.33)	0.97 (-0.50, 2.43)	0.50 (-0.70, 1.71)	0.39 (-0.76, 1.53)	0.16 (-0.95, 1.27)	1.04 (-0.37, 2.45)	0.13 (-1.56, 1.82)
		Superior frontal pole	0.09 (-1.12, 1.29)	0.43 (-1.04, 1.90)	0.82 (-0.64, 2.28)	0.19 (-1.01, 1.39)	0.17 (-0.97, 1.31)	0.76 (-0.31, 1.84)	-0.44 (-1.87, 0.98)	0.85 (-0.81, 2.51)
		Broca/Broadmann	-0.44 (-2.10, 1.23)	-0.29 (-2.33, 1.76)	-0.05 (-2.11, 2.01)	-0.25 (-1.92, 1.41)	-0.10 (-1.68, 1.48)	0.16 (-1.36, 1.69)	0.03 (-1.95, 2.01)	-1.81 (-4.07, 0.45)
		Dorsolateral prefrontal	0.00 (-1.12, 1.11)	0.99 (-0.35, 2.32)	1.11 (-0.23, 2.45)	1.11 (0.05, 2.18)*	0.60 (-0.44, 1.64)	1.26 (0.31, 2.21)*	-0.10 (-1.42, 1.23)	-0.11 (-1.66, 1.45)
No-Go vs. Go (n = 48)	L	Inferior frontal pole	1.04 (-1.33, 3.40)	-0.32 (-3.24, 2.60)	-0.37 (-3.31, 2.57)	-0.28 (-2.66, 2.10)	-0.12 (-2.38, 2.14)	0.68 (-1.49, 2.85)	-0.15 (-2.98, 2.68)	0.76 (-2.56, 4.07)
		Superior frontal pole	-0.11 (-2.13, 1.92)	-1.98 (-4.39, 0.43)	-1.82 (-4.26, 0.62)	-0.95 (-2.95, 1.05)	-0.26 (-2.18, 1.66)	0.57 (-1.27, 2.42)	-0.53 (-2.93, 1.87)	0.73 (-2.09, 3.55)
		Broca/Broadmann	-0.86 (-3.25, 1.52)	-0.13 (-3.07, 2.81)	0.44 (-2.51, 3.40)	-1.27 (-3.63, 1.09)	-0.12 (-2.39, 2.15)	-0.15 (-2.35, 2.04)	-0.03 (-2.87, 2.82)	0.76 (-2.58, 4.09)
		Dorsolateral prefrontal	1.02 (-1.10, 3.13)	2.08 (-0.46, 4.62)	1.84 (-0.73, 4.42)	-0.84 (-2.96, 1.27)	0.10 (-1.93, 2.12)	1.35 (-0.56, 3.26)	-2.61 (-5.02, -0.20)*	0.85 (-2.11, 3.82)
	R	Inferior frontal pole	0.38 (-1.72, 2.48)	0.49 (-2.09, 3.06)	0.57 (-2.02, 3.16)	-0.37 (-2.47, 1.73)	0.73 (-1.25, 2.72)	0.59 (-1.33, 2.51)	-0.33 (-2.83, 2.17)	-0.55 (-3.48, 2.39)
		Superior frontal pole	-0.73 (-3.27, 1.82)	-1.66 (-4.75, 1.43)	-2.06 (-5.15, 1.03)	-1.56 (-4.07, 0.95)	0.01 (-2.42, 2.43)	-0.38 (-2.72, 1.95)	-0.84 (-3.87, 2.18)	0.42 (-3.14, 3.99)
		Broca/Broadmann	-0.57 (-3.60, 2.46)	0.54 (-3.17, 4.26)	0.97 (-2.76, 4.70)	-1.11 (-4.12, 1.90)	-1.86 (-4.68, 0.96)	0.69 (-2.08, 3.46)	0.05 (-3.55, 3.66)	-0.90 (-5.13, 3.32)
		Dorsolateral prefrontal	-0.92 (-3.53, 1.69)	-0.24 (-3.45, 2.98)	-0.09 (-3.33, 3.14)	-1.50 (-4.08, 1.08)	-1.46 (-3.91, 0.99)	-0.09 (-2.49, 2.31)	-0.82 (-3.92, 2.29)	-0.59 (-4.25, 3.06)

Matching vs. control (Wisconsin Card Sort test) (*n* = 41)	L	Inferior frontal pole	-0.07 (-2.20, 2.06)	0.58 (-2.20, 3.35)	-0.15 (-2.98, 2.67)	1.33 (-0.88, 3.54)	1.01 (-1.23, 3.25)	-0.10 (-2.36, 2.17)	-0.98 (-3.42, 1.46)	1.01 (-1.94, 3.96)
		Superior frontal pole	-1.09 (-3.66, 1.48)	-3.11 (-6.34, 0.11)	-2.45 (-5.80, 0.89)	-0.62 (-3.35, 2.12)	0.05 (-2.71, 2.81)	-0.14 (-2.90, 2.62)	-0.76 (-3.76, 2.23)	-0.51 (-4.12, 3.11)
		Broca/Broadmann	-0.58 (-2.81, 1.64)	-0.83 (-3.73, 2.07)	-1.79 (-4.69, 1.10)	-0.77 (-3.12, 1.57)	0.19 (-2.19, 2.56)	-2.15 (-4.41, 0.11)	0.98 (-1.58, 3.54)	-1.40 (-4.48, 1.67)
		Dorsolateral prefrontal	0.09 (-2.19, 2.37)	-0.76 (-3.73, 2.20)	-1.21 (-4.21, 1.78)	-0.13 (-2.53, 2.28)	-0.36 (-2.78, 2.06)	-0.12 (-2.54, 2.30)	0.20 (-2.43, 2.83)	0.95 (-2.21, 4.11)
	R	Inferior frontal pole	0.04 (-2.88, 2.97)	0.99 (-2.81, 4.80)	-0.32 (-4.20, 3.56)	1.12 (-1.95, 4.19)	0.53 (-2.57, 3.64)	1.97 (-1.07, 5.01)	-2.10 (-5.41, 1.21)	5.45 (1.80, 9.09)*†
		Superior frontal pole	1.07 (-1.65, 3.79)	0.98 (-2.59, 4.55)	-0.10 (-3.74, 3.54)	1.35 (-1.51, 4.22)	0.31 (-2.60, 3.23)	0.89 (-2.01, 3.80)	-0.55 (-3.72, 2.62)	1.98 (-1.78, 5.75)
		Broca/Broadmann	-0.48 (-3.34, 2.39)	-0.48 (-4.22, 3.26)	-1.35 (-5.13, 2.43)	0.27 (-2.76, 3.30)	1.93 (-1.05, 4.91)	-1.39 (-4.41, 1.62)	0.17 (-3.14, 3.49)	-2.23 (-6.16, 1.70)
		Dorsolateral prefrontal	-0.49 (-3.27, 2.29)	2.54 (-1.00, 6.07)	1.84 (-1.80, 5.48)	0.74 (-2.19, 3.67)	2.16 (-0.71, 5.03)	0.20 (-2.76, 3.16)	-0.36 (-3.58, 2.86)	-0.47 (-4.35, 3.40)

Abbreviations: fNIRS, functional Near-Infrared Spectroscopy; n, number of participants; L, left; R, right; ETU, ethylenethiourea; PTU, propylenethiourea; TCPy, 3,5,6-trichloro-2-pyridinol; 3-PBA, 3-phenoxybenzoic acid; DCCA, 3-(2,2-dichlorovinyl)-2,2-dimethylcyclopropanecarboxylic acid; 2,4-D, 2,4-dichlorophenoxyacetic acid; TEB-OH, hydroxy-tebuconazole; GLY, glyphosate.
Models adjusted for age (continuous variable) and education level (≤6th grade, 7-11th grade).
*non-FDR corrected $p<0.05$, † FDR-corrected $p<0.05$.

Table S7. Adjusted associations [β (95% CI)] for a two-fold increase in urinary pesticide biomarker (specific gravity-adjusted) concentrations (imputed) with test performance outcomes on the three tasks administered during the fNIRS assessment in farmworkers from the Zarcero County, Costa Rica.

**Domain/Task**	***Urinary pesticide biomarkers***							
	**Insecticides**			**Fungicides**			**Herbicides**	
	**TCPy**	**3-PBA**	**DCCA**	**ETU**	**PTU**	**TEB-OH**	**2,4-D**	**GLY**
Sternberg test (*n* = 48)								
Accuracy	0.01 (-0.01, 0.04)	-0.01 (-0.05, 0.02)	-0.01 (-0.04, 0.02)	0.02 (0.00, 0.05)	-0.01 (-0.04, 0.01)	0.02 (-0.01, 0.04)	0.01 (-0.03, 0.04)	0.01 (-0.03, 0.05)
Reaction time	0.06 (-0.02, 0.13)	-0.07 (-0.16, 0.02)	-0.06 (-0.15, 0.03)	0.05 (-0.02, 0.13)	-0.03 (-0.10, 0.04)	0.00 (-0.07, 0.08)	0.03 (-0.06, 0.12)	-0.04 (-0.15, 0.07)
Go/No-Go (*n* = 48)								
Errors of omission	1.50 (-3.41, 6.40)	-0.20 (-6.23, 5.84)	-0.17 (-6.24, 5.91)	-1.28 (-6.18, 3.62)	0.75 (-3.91, 5.42)	-0.03 (-4.53, 4.48)	0.79 (-5.05, 6.64)	-0.80 (-7.66, 6.07)
Errors of commission	-0.50 (-1.01, 0.01)	-0.31 (-0.96, 0.33)	-0.26 (-0.91, 0.39)	-0.21 (-0.73, 0.32)	-0.30 (-0.80, 0.20)	-0.13 (-0.62, 0.35)	0.19 (-0.44, 0.82)	-0.16 (-0.90, 0.58)
Go reaction time	-0.01 (-0.03, 0.00)	-0.01 (-0.04, 0.01)	-0.02 (-0.04, 0.00)	-0.01 (-0.03, 0.00)	-0.02 (-0.03, 0.00)*	-0.01 (-0.03, 0.00)	0.01 (-0.02, 0.03)	0.01 (-0.01, 0.04)
No-Go reaction time	-0.02 (-0.03, 0.00)	-0.01 (-0.04, 0.01)	-0.02 (-0.04, 0.01)	-0.01 (-0.03, 0.00)	-0.02 (-0.04, 0.00)*	-0.01 (-0.03, 0.01)	0.01 (-0.02, 0.03)	0.02 (-0.01, 0.04)
D prime	-0.07 (-0.33, 0.19)	0.03 (-0.29, 0.35)	-0.08 (-0.41, 0.24)	0.02 (-0.24, 0.28)	-0.14 (-0.38, 0.11)	-0.07 (-0.31, 0.17)	0.15 (-0.16, 0.46)	0.30 (-0.05, 0.66)
Wisconsin Card Sort Test (*n* = 41)								
Total errors	-0.90 (-4.86, 3.06)	-1.16 (-6.33, 4.00)	0.77 (-4.49, 6.02)	-0.18 (-4.37, 4.01)	1.42 (-2.78, 5.61)	1.62 (-2.57, 5.80)	0.60 (-3.99, 5.18)	-1.79 (-7.29, 3.70)
Perseverative errors	-1.21 (-2.88, 0.47)	-1.29 (-3.49, 0.92)	-1.06 (-3.32, 1.19)	-0.73 (-2.53, 1.08)	1.62 (-0.13, 3.37)	-0.81 (-2.62, 1.00)	-0.11 (-2.10, 1.88)	-0.83 (-3.22, 1.55)

Abbreviations: fNIRS, functional Near-Infrared Spectroscopy; n, number of participants; ETU, ethylenethiourea; PTU, propylenethiourea; TCPy, 3,5,6-trichloro-2-pyridinol; 3-PBA, 3-phenoxybenzoic acid; DCCA, 3-(2,2-dichlorovinyl)-2,2-dimethylcyclopropanecarboxylic acid; 2,4-D, 2,4-dichlorophenoxyacetic acid; TEB-OH, hydroxy-tebuconazole; GLY, glyphosate.
Models adjusted for age (continuous variable) and education level (≤6th grade, 7-11th grade).
*non-FDR corrected p<0.05.

Table S8. Adjusted associations [β (95% CI)] for a two-fold increase in urinary pesticide biomarker (specific gravity-adjusted) concentrations (imputed) with fNIRS brain activation (HbO) by task and region of interest in farmworkers from the Zarcero County, Costa Rica (**excluding left-handed participants**).

Contrast	Hemisphere	Position	***Urinary pesticide biomarkers***							
			**Insecticides**			**Fungicides**			**Herbicides**	
			**TCPy**	**3-PBA**	**DCCA**	**ETU**	**PTU**	**TEB-OH**	**2,4-D**	**GLY**
Encoding vs. recall (Sternberg test) (*n* = 45)	L	Inferior frontal pole	-1.91 (-3.79, -0.03)*	-2.04 (-4.30, 0.22)	-1.81 (-4.10, 0.49)	-0.73 (-2.65, 1.19)	0.16 (-1.73, 2.05)	-1.71 (-3.41, 0.00)*	-0.87 (-3.14, 1.40)	-0.04 (-2.73, 2.64)
		Superior frontal pole	-1.68 (-3.25, -0.10)*	-2.29 (-4.12, -0.45)*	-2.31 (-4.15, -0.46)*	-0.65 (-2.27, 0.96)	0.00 (-1.58, 1.59)	-1.09 (-2.55, 0.37)	-1.40 (-3.27, 0.47)	0.93 (-1.31, 3.16)
		Broca/Broadmann	-1.23 (-2.99, 0.53)	-1.85 (-3.91, 0.21)	-1.45 (-3.56, 0.65)	-0.24 (-2.01, 1.52)	0.64 (-1.07, 2.36)	-1.08 (-2.68, 0.51)	-0.78 (-2.85, 1.29)	-0.07 (-2.52, 2.38)
		Dorsolateral prefrontal	-2.41 (-4.12, -0.70)*†	-3.11 (-5.10, -1.11)*†	-3.08 (-5.10, -1.07)*†	-1.20 (-2.99, 0.60)	-0.39 (-2.18, 1.40)	-1.58 (-3.20, 0.04)	-1.39 (-3.51, 0.73)	0.30 (-2.24, 2.84)
	R	Inferior frontal pole	-0.76 (-2.78, 1.27)	-2.42 (-4.72, -0.12)*	-2.34 (-4.67, -0.01)*	-0.48 (-2.47, 1.51)	0.65 (-1.30, 2.59)	-2.07 (-3.80, -0.35)*	-0.09 (-2.45, 2.26)	-0.32 (-3.09, 2.45)
		Superior frontal pole	-1.05 (-2.72, 0.62)	-1.91 (-3.85, 0.02)*	-2.03 (-3.97, -0.10)*	-0.18 (-1.84, 1.49)	0.21 (-1.42, 1.84)	-1.24 (-2.73, 0.25)	-0.80 (-2.75, 1.15)	-0.11 (-2.42, 2.21)
		Broca/Broadmann	-0.73 (-2.52, 1.06)	-2.28 (-4.30, -0.26)*	-2.14 (-4.19, -0.09)*	-0.25 (-2.01, 1.52)	0.22 (-1.50, 1.95)	-1.32 (-2.90, 0.26)	0.01 (-2.08, 2.09)	-0.46 (-2.91, 1.99)
		Dorsolateral prefrontal	-1.16 (-3.11, 0.78)	-2.34 (-4.58, -0.11)*	-2.10 (-4.38, 0.18)	-0.78 (-2.70, 1.15)	0.32 (-1.57, 2.22)	-1.43 (-3.17, 0.31)	-0.64 (-2.92, 1.64)	0.11 (-2.59, 2.80)
No-Go vs. Go (*n* = 45)	L	Inferior frontal pole	-0.22 (-2.36, 1.92)	-1.49 (-3.99, 1.01)	-1.13 (-3.67, 1.41)	0.79 (-1.29, 2.87)	0.33 (-1.72, 2.37)	-0.15 (-2.09, 1.79)	0.21 (-2.27, 2.68)	0.89 (-2.01, 3.78)
		Superior frontal pole	-0.88 (-3.38, 1.62)	-2.46 (-5.35, 0.43)	-2.51 (-5.42, 0.39)	-0.44 (-2.90, 2.02)	-1.57 (-3.93, 0.80)	-0.69 (-2.96, 1.57)	-0.08 (-2.99, 2.83)	1.02 (-2.39, 4.43)
		Broca/Broadmann	-0.60 (-2.90, 1.71)	-1.75 (-4.44, 0.94)	-1.58 (-4.30, 1.14)	0.49 (-1.76, 2.75)	0.15 (-2.07, 2.36)	-1.06 (-3.12, 1.01)	0.62 (-2.04, 3.29)	0.75 (-2.39, 3.88)
		Dorsolateral prefrontal	-1.26 (-3.52, 0.99)	-2.02 (-4.66, 0.62)	-1.73 (-4.41, 0.95)	0.34 (-1.90, 2.58)	-0.41 (-2.60, 1.77)	0.01 (-2.06, 2.08)	-0.77 (-3.40, 1.87)	0.53 (-2.58, 3.64)
	R	Inferior frontal pole	-0.07 (-1.75, 1.61)	0.27 (-1.72, 2.27)	0.09 (-1.92, 2.10)	0.34 (-1.30, 1.99)	0.36 (-1.25, 1.96)	0.25 (-1.27, 1.77)	-0.59 (-2.52, 1.35)	1.48 (-0.76, 3.72)
		Superior frontal pole	-0.60 (-2.55, 1.35)	-0.67 (-3.00, 1.65)	-0.68 (-3.02, 1.66)	0.01 (-1.91, 1.93)	-0.78 (-2.64, 1.08)	-0.29 (-2.07, 1.48)	-0.59 (-2.85, 1.67)	-0.19 (-2.86, 2.47)
		Broca/Broadmann	-1.19 (-3.15, 0.78)	-0.70 (-3.07, 1.67)	-0.79 (-3.17, 1.60)	-0.21 (-2.17, 1.74)	-0.52 (-2.43, 1.39)	-0.20 (-2.00, 1.61)	-0.53 (-2.84, 1.78)	1.33 (-1.36, 4.01)
		Dorsolateral prefrontal	-1.88 (-3.78, 0.03)*	-0.82 (-3.17, 1.54)	-0.92 (-3.29, 1.44)	-1.42 (-3.32, 0.48)	-1.73 (-3.56, 0.10)	-1.03 (-2.80, 0.75)	-1.22 (-3.49, 1.05)	-0.91 (-3.61, 1.79)
Matching vs. control	L	Inferior frontal pole	-1.95 (-5.15, 1.25)	-1.82 (-5.91, 2.28)	-2.76 (-6.85, 1.34)	-1.66 (-5.01, 1.69)	-1.27 (-4.80, 2.26)	-1.49 (-4.91, 1.94)	0.28 (-3.41, 3.97)	-1.06 (-5.51, 3.40)

(Wisconsin Card Sort test) (*n* = 38)		Superior frontal pole	-1.64 (-4.37, 1.09)	-2.20 (-5.65, 1.25)	-2.36 (-5.85, 1.13)	-1.63 (-4.47, 1.21)	-0.39 (-3.42, 2.64)	0.08 (-2.88, 3.03)	0.41 (-2.74, 3.56)	-1.27 (-5.05, 2.51)
		Broca/Broadmann	-0.94 (-4.07, 2.19)	-1.33 (-5.29, 2.64)	-1.89 (-5.89, 2.10)	-2.47 (-5.63, 0.69)	-2.48 (-5.79, 0.84)	-1.34 (-4.65, 1.97)	0.96 (-2.59, 4.50)	-1.76 (-6.02, 2.50)
		Dorsolateral prefrontal	-0.75 (-3.41, 1.92)	-2.52 (-5.80, 0.76)	-2.33 (-5.68, 1.01)	-1.95 (-4.65, 0.75)	-0.96 (-3.85, 1.94)	-0.63 (-3.46, 2.20)	0.19 (-2.84, 3.21)	-1.44 (-5.06, 2.19)
	R	Inferior frontal pole	-1.36 (-4.29, 1.57)	-1.38 (-5.11, 2.36)	-2.10 (-5.85, 1.65)	-0.83 (-3.91, 2.24)	-0.80 (-4.02, 2.42)	-0.81 (-3.95, 2.33)	0.50 (-2.85, 3.86)	0.13 (-3.93, 4.19)
		Superior frontal pole	-0.69 (-3.53, 2.14)	-1.12 (-4.70, 2.47)	-1.37 (-5.00, 2.25)	0.52 (-2.44, 3.47)	-0.88 (-3.96, 2.20)	0.63 (-2.38, 3.64)	0.69 (-2.52, 3.89)	0.81 (-3.06, 4.69)
		Broca/Broadmann	-0.03 (-2.90, 2.84)	-0.51 (-4.15, 3.12)	-1.29 (-4.95, 2.38)	-0.64 (-3.62, 2.33)	0.29 (-2.84, 3.41)	-0.37 (-3.41, 2.67)	0.77 (-2.47, 4.00)	1.22 (-2.68, 5.12)
		Dorsolateral prefrontal	-1.18 (-3.69, 1.33)	-0.71 (-3.92, 2.50)	-1.21 (-4.46, 2.03)	-0.32 (-2.96, 2.32)	-1.28 (-4.01, 1.45)	-0.06 (-2.75, 2.64)	1.67 (-1.14, 4.49)	-0.81 (-4.27, 2.66)

Abbreviations: fNIRS, functional Near-Infrared Spectroscopy; n, number of participants; L, left; R, right; ETU, ethylenethiourea; PTU, propylenethiourea; TCPy, 3,5,6-trichloro-2-pyridinol; 3-PBA, 3-phenoxybenzoic acid; DCCA, 3-(2,2-dichlorovinyl)-2,2-dimethylcyclopropanecarboxylic acid; 2,4-D, 2,4-dichlorophenoxyacetic acid; TEB-OH, hydroxy-tebuconazole; GLY, glyphosate.
Models adjusted for age (continuous variable) and education level (≤6th grade, 7-11th grade).
*non-FDR corrected p<0.05, † FDR-corrected p<0.05.

Table S9. Adjusted associations [β (95% CI)] for a two-fold increase in urinary pesticide biomarker (specific gravity-adjusted) concentrations (imputed) with fNIRS brain activation (HbO) by task and region of interest in farmworkers from the Zarcero County, Costa Rica (**excluding female participants**).

**Contrast**	**Hemisphere**	**Position**	***Urinary pesticide biomarkers***							
			**Insecticides**			**Fungicides**			**Herbicides**	
			**TCPy**	**3-PBA**	**DCCA**	**ETU**	**PTU**	**TEB-OH**	**2,4-D**	**GLY**
Encoding vs. recall (Sternberg test) (*n* = 46)	L	Inferior frontal pole	-1.97 (-3.73, -0.21)*	-2.23 (-4.42, -0.05)*†	-2.01 (-4.25, 0.23)	-0.64 (-2.48, 1.21)	0.24 (-1.52, 2.01)	-1.81 (-3.42, -0.19)*	-0.96 (-3.16, 1.25)	0.49 (-2.19, 3.17)
		Superior frontal pole	-1.69 (-3.21, -0.17)*	-2.36 (-4.19, -0.52)*†	-2.30 (-4.17, -0.43)*†	-0.54 (-2.13, 1.05)	-0.05 (-1.57, 1.48)	-1.05 (-2.49, 0.39)	-1.35 (-3.22, 0.52)	1.26 (-1.02, 3.54)
		Broca/Broadmann	-1.28 (-2.94, 0.39)	-1.97 (-3.99, 0.04)*†	-1.59 (-3.67, 0.49)	-0.12 (-1.82, 1.59)	0.86 (-0.74, 2.46)	-1.22 (-2.75, 0.31)	-0.80 (-2.83, 1.22)	0.43 (-2.04, 2.89)
		Dorsolateral prefrontal	-2.33 (-3.97, -0.70)*†	-3.17 (-5.14, -1.21)*†	-3.17 (-5.16, -1.17)*†	-1.10 (-2.85, 0.64)	-0.28 (-1.97, 1.41)	-1.60 (-3.16, -0.03)*	-1.39 (-3.48, 0.70)	0.56 (-2.01, 3.13)
	R	Inferior frontal pole	-0.83 (-2.77, 1.11)	-2.50 (-4.77, -0.22)*†	-2.35 (-4.68, -0.02)*	-0.28 (-2.23, 1.67)	0.75 (-1.09, 2.59)	-2.07 (-3.74, -0.39)*	-0.02 (-2.36, 2.31)	0.18 (-2.63, 3.00)
		Superior frontal pole	-1.13 (-2.66, 0.40)	-2.02 (-3.84, -0.20)*†	-2.12 (-3.96, -0.27)*†	-0.07 (-1.63, 1.49)	0.30 (-1.18, 1.78)	-1.34 (-2.72, 0.04)	-0.82 (-2.68, 1.03)	0.44 (-1.81, 2.69)
		Broca/Broadmann	-0.81 (-2.48, 0.85)	-2.42 (-4.35, -0.49)*†	-2.32 (-4.29, -0.34)*†	-0.22 (-1.89, 1.46)	0.28 (-1.32, 1.87)	-1.43 (-2.92, 0.05)	-0.09 (-2.11, 1.92)	-0.03 (-2.46, 2.40)
		Dorsolateral prefrontal	-1.24 (-3.07, 0.59)	-2.51 (-4.68, -0.35)*†	-2.30 (-4.52, -0.08)*	-0.64 (-2.50, 1.21)	0.52 (-1.25, 2.28)	-1.55 (-3.20, 0.09)	-0.71 (-2.93, 1.51)	0.69 (-2.00, 3.38)
No-Go vs. Go (*n* = 46)	L	Inferior frontal pole	-0.20 (-2.24, 1.85)	-1.42 (-3.89, 1.06)	-0.98 (-3.52, 1.55)	0.76 (-1.26, 2.78)	0.12 (-1.82, 2.06)	-0.01 (-1.89, 1.86)	0.32 (-2.12, 2.76)	0.68 (-2.26, 3.62)
		Superior frontal pole	-0.95 (-3.38, 1.47)	-2.46 (-5.37, 0.45)	-2.39 (-5.35, 0.57)	-0.33 (-2.76, 2.11)	-1.52 (-3.79, 0.75)	-0.58 (-2.82, 1.66)	0.10 (-2.81, 3.02)	0.95 (-2.56, 4.46)
		Broca/Broadmann	-0.49 (-2.66, 1.69)	-1.61 (-4.25, 1.02)	-1.36 (-4.05, 1.32)	0.45 (-1.71, 2.62)	-0.15 (-2.22, 1.91)	-0.84 (-2.83, 1.14)	0.74 (-1.85, 3.33)	0.52 (-2.61, 3.66)
		Dorsolateral prefrontal	-0.97 (-3.20, 1.27)	-1.95 (-4.65, 0.76)	-1.59 (-4.36, 1.18)	0.43 (-1.81, 2.68)	-0.64 (-2.77, 1.49)	0.34 (-1.73, 2.41)	-0.69 (-3.37, 1.99)	0.67 (-2.58, 3.91)
	R	Inferior frontal pole	-0.09 (-1.69, 1.51)	0.34 (-1.63, 2.31)	0.27 (-1.73, 2.27)	0.39 (-1.20, 1.99)	0.20 (-1.32, 1.72)	0.34 (-1.13, 1.81)	-0.47 (-2.38, 1.44)	1.54 (-0.72, 3.81)
		Superior frontal pole	-0.57 (-2.52, 1.37)	-0.71 (-3.10, 1.67)	-0.62 (-3.04, 1.80)	0.09 (-1.85, 2.03)	-0.93 (-2.75, 0.90)	-0.12 (-1.91, 1.67)	-0.54 (-2.86, 1.78)	-0.04 (-2.85, 2.77)
		Broca/Broadmann	-0.97 (-2.87, 0.93)	-0.62 (-2.98, 1.74)	-0.75 (-3.14, 1.63)	-0.25 (-2.17, 1.66)	-0.56 (-2.38, 1.25)	-0.11 (-1.87, 1.66)	-0.55 (-2.84, 1.74)	1.30 (-1.44, 4.04)
		Dorsolateral prefrontal	-1.56 (-3.41, 0.29)	-0.69 (-3.03, 1.66)	-0.80 (-3.17, 1.58)	-1.47 (-3.32, 0.39)	-1.91 (-3.63, -0.20)*	-0.74 (-2.49, 1.00)	-1.19 (-3.45, 1.06)	-1.32 (-4.05, 1.40)

Matching vs. control (Wisconsin Card Sort test) (*n* = 39)	L	Inferior frontal pole	-1.79 (-4.90, 1.32)	-1.81 (-5.92, 2.29)	-3.03 (-7.14, 1.09)	-2.12 (-5.40, 1.15)	-1.68 (-5.02, 1.66)	-1.44 (-4.79, 1.91)	-0.02 (-3.73, 3.68)	-2.10 (-6.64, 2.43)
		Superior frontal pole	-1.45 (-4.11, 1.21)	-2.31 (-5.76, 1.15)	-2.78 (-6.28, 0.71)	-1.92 (-4.71, 0.86)	-0.69 (-3.57, 2.20)	-0.08 (-2.97, 2.81)	-0.19 (-3.35, 2.97)	-1.17 (-5.07, 2.72)
		Broca/Broadmann	-0.74 (-3.87, 2.38)	-1.38 (-5.46, 2.69)	-2.35 (-6.47, 1.76)	-2.96 (-6.11, 0.19)	-2.76 (-5.98, 0.45)	-1.37 (-4.69, 1.94)	0.36 (-3.30, 4.02)	-2.44 (-6.89, 2.02)
		Dorsolateral prefrontal	-0.39 (-3.06, 2.27)	-2.56 (-5.94, 0.82)	-2.72 (-6.17, 0.72)	-2.28 (-4.99, 0.43)	-1.46 (-4.27, 1.35)	-0.49 (-3.33, 2.36)	-0.43 (-3.55, 2.68)	-1.41 (-5.24, 2.42)
	R	Inferior frontal pole	-1.41 (-4.41, 1.59)	-1.48 (-5.42, 2.46)	-2.45 (-6.42, 1.53)	-1.39 (-4.56, 1.79)	-1.42 (-4.63, 1.80)	-0.97 (-4.20, 2.26)	0.01 (-3.54, 3.56)	-0.58 (-4.97, 3.82)
		Superior frontal pole	-0.59 (-3.37, 2.18)	-1.07 (-4.69, 2.55)	-1.46 (-5.14, 2.23)	0.15 (-2.78, 3.09)	-1.31 (-4.25, 1.63)	0.63 (-2.33, 3.59)	0.47 (-2.77, 3.71)	0.35 (-3.67, 4.37)
		Broca/Broadmann	-0.08 (-2.95, 2.79)	-0.65 (-4.39, 3.10)	-1.67 (-5.46, 2.12)	-1.07 (-4.08, 1.94)	-0.28 (-3.35, 2.78)	-0.55 (-3.60, 2.51)	0.19 (-3.16, 3.54)	1.12 (-3.02, 5.25)
		Dorsolateral prefrontal	-0.85 (-3.35, 1.64)	-0.73 (-4.01, 2.54)	-1.52 (-4.84, 1.80)	-0.66 (-3.30, 1.99)	-1.71 (-4.33, 0.91)	0.02 (-2.66, 2.70)	1.11 (-1.79, 4.02)	-0.69 (-4.32, 2.94)

Abbreviations: fNIRS, functional Near-Infrared Spectroscopy; n, number of participants; L, left; R, right; ETU, ethylenethiourea; PTU, propylenethiourea; TCPy, 3,5,6-trichloro-2-pyridinol; 3-PBA, 3-phenoxybenzoic acid; DCCA, 3-(2,2-dichlorovinyl)-2,2-dimethylcyclopropanecarboxylic acid; 2,4-D, 2,4-dichlorophenoxyacetic acid; TEB-OH, hydroxy-tebuconazole; GLY, glyphosate.

Models adjusted for age (continuous variable) and education level (≤6th grade, 7-11th grade).

*non-FDR corrected p<0.05, † FDR-corrected p<0.05.

Table S10. Adjusted associations [β (95% CI)] for a two-fold increase in urinary pesticide biomarker (specific gravity-adjusted) concentrations (imputed) with fNIRS brain activation (HbO) by task and region of interest in farmworkers from the Zarcero County, Costa Rica (**excluding participant with neurological disorders**).

**Contrast**	**Hemisphere**	**Position**	***Urinary pesticide biomarkers***							
			**Insecticides**			**Fungicides**			**Herbicides**	
			**TCPy**	**3-PBA**	**DCCA**	**ETU**	**PTU**	**TEB-OH**	**2,4-D**	**GLY**
Encoding vs. recall (Sternberg test) (n = 47)	L	Inferior frontal pole	-1.90 (-3.68, -0.12)*	-2.11 (-4.32, 0.10)	-1.82 (-4.07, 0.43)	-0.62 (-2.49, 1.24)	0.27 (-1.54, 2.07)	-1.66 (-3.30, -0.03)*	-0.78 (-2.99, 1.44)	0.07 (-2.55, 2.69)
		Superior frontal pole	-1.66 (-3.18, -0.15)*	-2.36 (-4.18, -0.53)*†	-2.30 (-4.14, -0.45)*	-0.54 (-2.13, 1.04)	0.01 (-1.53, 1.55)	-1.01 (-2.44, 0.41)	-1.30 (-3.16, 0.55)	1.06 (-1.16, 3.27)
		Broca/Broadmann	-1.20 (-2.88, 0.47)	-1.81 (-3.85, 0.22)	-1.37 (-3.46, 0.72)	-0.14 (-1.86, 1.58)	0.81 (-0.84, 2.45)	-1.09 (-2.63, 0.45)	-0.67 (-2.70, 1.36)	-0.02 (-2.43, 2.39)
		Dorsolateral prefrontal	-2.30 (-3.92, -0.68)*†	-3.12 (-5.07, -1.17)*†	-3.07 (-5.05, -1.09)*†	-1.12 (-2.85, 0.62)	-0.30 (-2.00, 1.41)	-1.53 (-3.08, 0.02)*	-1.33 (-3.39, 0.74)	0.35 (-2.13, 2.82)
	R	Inferior frontal pole	-0.77 (-2.71, 1.17)	-2.41 (-4.70, -0.13)*	-2.24 (-4.56, 0.08)	-0.31 (-2.26, 1.64)	0.74 (-1.13, 2.62)	-1.96 (-3.65, -0.28)*	0.04 (-2.28, 2.36)	-0.17 (-2.91, 2.57)
		Superior frontal pole	-1.05 (-2.63, 0.53)	-1.92 (-3.82, -0.03)*	-2.00 (-3.90, -0.10)*	-0.11 (-1.72, 1.50)	0.30 (-1.26, 1.85)	-1.22 (-2.65, 0.21)	-0.73 (-2.63, 1.18)	-0.06 (-2.32, 2.20)
		Broca/Broadmann	-0.72 (-2.39, 0.95)	-2.21 (-4.17, -0.25)*	-2.05 (-4.04, -0.05)*	-0.29 (-1.97, 1.40)	0.10 (-1.52, 1.73)	-1.29 (-2.79, 0.20)	-0.01 (-2.01, 2.00)	-0.58 (-2.93, 1.78)
		Dorsolateral prefrontal	-1.14 (-2.99, 0.70)	-2.29 (-4.48, -0.09)*	-1.98 (-4.22, 0.26)	-0.69 (-2.55, 1.18)	0.37 (-1.44, 2.17)	-1.39 (-3.06, 0.27)	-0.56 (-2.78, 1.66)	0.11 (-2.51, 2.74)
No-Go vs. Go (n = 47)	L	Inferior frontal pole	-0.25 (-2.28, 1.78)	-1.56 (-4.01, 0.89)	-1.18 (-3.67, 1.31)	0.78 (-1.23, 2.80)	0.24 (-1.71, 2.20)	-0.11 (-1.96, 1.75)	0.21 (-2.20, 2.62)	0.95 (-1.88, 3.77)
		Superior frontal pole	-1.03 (-3.39, 1.34)	-2.66 (-5.48, 0.15)	-2.62 (-5.46, 0.22)	-0.24 (-2.62, 2.14)	-1.29 (-3.55, 0.98)	-0.66 (-2.84, 1.51)	0.08 (-2.76, 2.91)	1.32 (-1.99, 4.64)
		Broca/Broadmann	-0.53 (-2.72, 1.66)	-1.79 (-4.43, 0.85)	-1.64 (-4.30, 1.03)	0.44 (-1.75, 2.63)	-0.04 (-2.15, 2.08)	-0.94 (-2.92, 1.04)	0.57 (-2.03, 3.16)	0.75 (-2.32, 3.81)
		Dorsolateral prefrontal	-1.00 (-3.20, 1.20)	-2.07 (-4.72, 0.59)	-1.74 (-4.44, 0.95)	0.47 (-1.74, 2.68)	-0.50 (-2.63, 1.64)	0.29 (-1.74, 2.32)	-0.72 (-3.34, 1.91)	0.82 (-2.27, 3.92)
	R	Inferior frontal pole	-0.11 (-1.71, 1.48)	0.21 (-1.76, 2.17)	0.07 (-1.91, 2.05)	0.40 (-1.19, 1.99)	0.34 (-1.20, 1.88)	0.28 (-1.18, 1.74)	-0.53 (-2.43, 1.36)	1.59 (-0.59, 3.78)
		Superior frontal pole	-0.62 (-2.46, 1.23)	-0.89 (-3.15, 1.37)	-0.83 (-3.11, 1.45)	0.19 (-1.66, 2.04)	-0.64 (-2.42, 1.14)	-0.15 (-1.84, 1.54)	-0.47 (-2.67, 1.72)	0.19 (-2.41, 2.78)
		Broca/Broadmann	-1.01 (-2.85, 0.83)	-0.79 (-3.07, 1.49)	-0.97 (-3.26, 1.32)	-0.19 (-2.05, 1.67)	-0.32 (-2.12, 1.47)	-0.15 (-1.86, 1.55)	-0.54 (-2.75, 1.67)	1.45 (-1.13, 4.02)
		Dorsolateral prefrontal	-1.64 (-3.44, 0.16)	-0.95 (-3.23, 1.33)	-1.13 (-3.42, 1.16)	-1.39 (-3.21, 0.43)	-1.67 (-3.40, 0.06)	-0.86 (-2.54, 0.83)	-1.26 (-3.45, 0.92)	-0.75 (-3.35, 1.86)
Matching vs. control	L	Inferior frontal pole	-1.83 (-4.89, 1.24)	-1.82 (-5.85, 2.21)	-2.93 (-6.96, 1.09)	-2.04 (-5.27, 1.18)	-1.64 (-4.92, 1.63)	-1.49 (-4.78, 1.79)	-0.08 (-3.69, 3.53)	-1.52 (-5.84, 2.80)

(Wisconsin Card Sort test) (*n* = 41)		Superior frontal pole	-1.41 (-4.05, 1.23)	-2.16 (-5.58, 1.26)	-2.53 (-5.98, 0.92)	-1.99 (-4.74, 0.76)	-0.83 (-3.67, 2.00)	0.08 (-2.77, 2.93)	0.05 (-3.04, 3.15)	-1.68 (-5.36, 2.01)
		Broca/Broadmann	-0.75 (-3.81, 2.31)	-1.29 (-5.27, 2.69)	-2.12 (-6.13, 1.88)	-2.94 (-6.02, 0.15)	-2.83 (-5.95, 0.29)	-1.30 (-4.53, 1.94)	0.48 (-3.06, 4.03)	-2.31 (-6.52, 1.89)
		Dorsolateral prefrontal	-0.36 (-2.99, 2.26)	-2.44 (-5.77, 0.89)	-2.52 (-5.90, 0.86)	-2.33 (-4.99, 0.33)	-1.56 (-4.31, 1.18)	-0.35 (-3.14, 2.44)	-0.23 (-3.27, 2.80)	-1.81 (-5.41, 1.80)
	R	Inferior frontal pole	-1.41 (-4.32, 1.50)	-1.45 (-5.27, 2.38)	-2.34 (-6.18, 1.49)	-1.37 (-4.46, 1.71)	-1.43 (-4.53, 1.67)	-0.95 (-4.07, 2.18)	0.04 (-3.37, 3.46)	-0.53 (-4.63, 3.58)
		Superior frontal pole	-0.60 (-3.29, 2.10)	-1.11 (-4.62, 2.39)	-1.52 (-5.06, 2.03)	0.16 (-2.69, 3.01)	-1.24 (-4.08, 1.60)	0.59 (-2.28, 3.45)	0.38 (-2.73, 3.50)	0.43 (-3.33, 4.18)
		Broca/Broadmann	-0.06 (-2.86, 2.75)	-0.55 (-4.20, 3.11)	-1.49 (-5.18, 2.19)	-1.11 (-4.05, 1.83)	-0.39 (-3.36, 2.59)	-0.43 (-3.41, 2.55)	0.35 (-2.89, 3.59)	0.64 (-3.25, 4.54)
		Dorsolateral prefrontal	-0.81 (-3.29, 1.66)	-0.64 (-3.88, 2.60)	-1.39 (-4.66, 1.87)	-0.73 (-3.35, 1.89)	-1.79 (-4.36, 0.79)	0.15 (-2.50, 2.79)	1.25 (-1.59, 4.10)	-1.18 (-4.62, 2.26)

Abbreviations: fNIRS, functional Near-Infrared Spectroscopy; n, number of participants; L, left; R, right; ETU, ethylenethiourea; PTU, propylenethiourea; TCPy, 3,5,6-trichloro-2-pyridinol; 3-PBA, 3-phenoxybenzoic acid; DCCA, 3-(2,2-dichlorovinyl)-2,2-dimethylcyclopropanecarboxylic acid; 2,4-D, 2,4-dichlorophenoxyacetic acid; TEB-OH, hydroxy-tebuconazole; GLY, glyphosate.
Models adjusted for age (continuous variable) and education level (≤6th grade, 7-11th grade).
*non-FDR corrected p<0.05, † FDR-corrected p<0.05.

Table S11. Adjusted associations [β (95% CI)] for a two-fold increase in urinary pesticide biomarker (specific gravity-adjusted) concentrations (imputed) with fNIRS brain activation (HbO) for Sternberg contrasts by region of interest in farmworkers from the Zarcero County, Costa Rica (**excluding participants who had outliers in task accuracy**).

Contrast	Hemisphere	Position	***Urinary pesticide biomarkers***							
			**Insecticides**			**Fungicides**			**Herbicides**	
			**TCPy**	**3-PBA**	**DCCA**	**ETU**	**PTU**	**TEB-OH**	**2,4-D**	**GLY**
Encoding vs. recall (n = 45)	L	Inferior frontal pole	-1.96 (-3.83, -0.09)*	-2.02 (-4.31, 0.27)	-1.72 (-4.05, 0.61)	-0.73 (-2.69, 1.22)	0.21 (-1.65, 2.07)	-1.96 (-3.73, -0.18)*	-0.80 (-3.06, 1.47)	-0.18 (-2.96, 2.59)
		Superior frontal pole	-1.78 (-3.35, -0.21)*	-2.23 (-4.11, -0.35)*	-2.15 (-4.05, -0.24)*	-0.73 (-2.38, 0.93)	-0.16 (-1.73, 1.41)	-1.11 (-2.66, 0.44)	-1.30 (-3.18, 0.59)	0.96 (-1.36, 3.29)
		Broca/Broadmann	-1.36 (-3.10, 0.37)	-1.72 (-3.80, 0.37)	-1.32 (-3.45, 0.81)	-0.32 (-2.11, 1.47)	0.88 (-0.78, 2.55)	-1.51 (-3.15, 0.13)	-0.70 (-2.75, 1.36)	-0.37 (-2.89, 2.14)
		Dorsolateral prefrontal	-2.49 (-4.17, -0.81)*†	-3.06 (-5.08, -1.05)*†	-3.04 (-5.08, -1.00)*†	-1.31 (-3.12, 0.49)	-0.30 (-2.05, 1.45)	-1.91 (-3.58, -0.25)*	-1.35 (-3.45, 0.75)	0.12 (-2.49, 2.73)
	R	Inferior frontal pole	-0.77 (-2.81, 1.27)	-2.55 (-4.90, -0.21)*	-2.36 (-4.74, 0.02)*	-0.22 (-2.28, 1.83)	0.83 (-1.09, 2.75)	-2.25 (-4.08, -0.43)*	0.04 (-2.34, 2.41)	-0.08 (-2.97, 2.81)
		Superior frontal pole	-1.22 (-2.86, 0.43)	-1.87 (-3.82, 0.09)	-1.96 (-3.91, 0.00)*	-0.26 (-1.95, 1.43)	0.21 (-1.38, 1.81)	-1.45 (-2.99, 0.10)	-0.73 (-2.67, 1.21)	-0.16 (-2.54, 2.22)
		Broca/Broadmann	-0.88 (-2.61, 0.85)	-2.38 (-4.36, -0.40)*	-2.31 (-4.31, -0.31)*	-0.30 (-2.05, 1.46)	0.47 (-1.17, 2.12)	-1.90 (-3.47, -0.34)*	-0.05 (-2.08, 1.98)	-0.75 (-3.21, 1.71)
		Dorsolateral prefrontal	-1.28 (-3.20, 0.65)	-2.29 (-4.55, -0.03)*	-2.04 (-4.34, 0.26)	-0.82 (-2.77, 1.14)	0.57 (-1.27, 2.42)	-1.89 (-3.67, -0.11)*	-0.58 (-2.85, 1.68)	-0.15 (-2.92, 2.62)

Abbreviations: fNIRS, functional Near-Infrared Spectroscopy; n, number of participants; L, left; R, right; ETU, ethylenethiourea; PTU, propylenethiourea; TCPy, 3,5,6-trichloro-2-pyridinol; 3-PBA, 3-phenoxybenzoic acid; DCCA, 3-(2,2-dichlorovinyl)-2,2-dimethylcyclopropanecarboxylic acid; 2,4-D, 2,4-dichlorophenoxyacetic acid; TEB-OH, hydroxy-tebuconazole; GLY, glyphosate.
Models adjusted for age (continuous variable) and education level (≤6th grade, 7-11th grade).
*non-FDR corrected p<0.05, † FDR-corrected p<0.05.

Table S12. Adjusted associations [β (95% CI)] for a two-fold increase in urinary pesticide biomarker (specific gravity-adjusted) concentrations (imputed) with fNIRS brain activation (HbO) for Go/No-Go contrast by region of interest in farmworkers from the Zarcero County, Costa Rica (**excluding participants who had outliers in errors of omission**).

Contrast	Hemisphere	Position	***Urinary pesticide biomarkers***							
			**Insecticides**			**Fungicides**			**Herbicides**	
			**TCPy**	**3-PBA**	**DCCA**	**ETU**	**PTU**	**TEB-OH**	**2,4-D**	**GLY**
No-Go vs. Go (*n* = 45)	L	Inferior frontal pole	0.33 (-1.93, 2.59)	-1.50 (-3.96, 0.96)	-1.05 (-3.55, 1.45)	0.96 (-1.11, 3.03)	-0.15 (-2.27, 1.96)	-0.18 (-2.06, 1.69)	0.37 (-2.07, 2.81)	0.76 (-2.10, 3.61)
		Superior frontal pole	-0.76 (-3.48, 1.96)	-2.51 (-5.43, 0.41)	-2.39 (-5.34, 0.57)	-0.20 (-2.72, 2.33)	-1.95 (-4.43, 0.53)	-0.69 (-2.94, 1.57)	0.12 (-2.83, 3.07)	1.08 (-2.36, 4.52)
		Broca/Broadmann	0.12 (-2.32, 2.55)	-1.80 (-4.44, 0.84)	-1.57 (-4.24, 1.10)	0.70 (-1.54, 2.94)	-0.30 (-2.57, 1.98)	-1.00 (-3.00, 0.99)	0.69 (-1.93, 3.31)	0.54 (-2.53, 3.62)
		Dorsolateral prefrontal	-0.58 (-3.07, 1.90)	-2.00 (-4.69, 0.68)	-1.61 (-4.34, 1.13)	0.64 (-1.66, 2.93)	-0.97 (-3.29, 1.34)	0.24 (-1.82, 2.31)	-0.63 (-3.32, 2.05)	0.57 (-2.58, 3.72)
	R	Inferior frontal pole	0.26 (-1.53, 2.06)	0.28 (-1.71, 2.26)	0.19 (-1.82, 2.19)	0.49 (-1.17, 2.14)	0.04 (-1.64, 1.72)	0.24 (-1.25, 1.72)	-0.44 (-2.38, 1.50)	1.46 (-0.76, 3.69)
		Superior frontal pole	-0.40 (-2.55, 1.75)	-0.65 (-3.02, 1.73)	-0.51 (-2.91, 1.88)	0.14 (-1.85, 2.13)	-1.42 (-3.38, 0.54)	-0.19 (-1.97, 1.59)	-0.41 (-2.73, 1.91)	-0.05 (-2.77, 2.67)
		Broca/Broadmann	-0.86 (-2.97, 1.24)	-0.69 (-3.03, 1.65)	-0.82 (-3.18, 1.53)	-0.19 (-2.16, 1.77)	-0.83 (-2.80, 1.14)	-0.18 (-1.94, 1.58)	-0.53 (-2.82, 1.75)	1.09 (-1.57, 3.76)
		Dorsolateral prefrontal	-1.59 (-3.69, 0.51)	-0.82 (-3.20, 1.55)	-0.94 (-3.33, 1.45)	-1.40 (-3.34, 0.55)	-2.25 (-4.15, -0.36)*	-0.85 (-2.62, 0.91)	-1.29 (-3.58, 1.01)	-0.96 (-3.67, 1.75)

Abbreviations: fNIRS, functional Near-Infrared Spectroscopy; n, number of participants; L, left; R, right; ETU, ethylenethiourea; PTU, propylenethiourea; TCPy, 3,5,6-trichloro-2-pyridinol; 3-PBA, 3-phenoxybenzoic acid; DCCA, 3-(2,2-dichlorovinyl)-2,2-dimethylcyclopropanecarboxylic acid; 2,4-D, 2,4-dichlorophenoxyacetic acid; TEB-OH, hydroxy-tebuconazole; GLY, glyphosate.
Models adjusted for age (continuous variable) and education level (≤6th grade, 7-11th grade).
*non-FDR corrected p<0.05, † FDR-corrected p<0.05.

Table S13. Adjusted associations [β (95% CI)] for a two-fold increase in urinary pesticide biomarker (specific gravity-adjusted) concentrations (imputed) with fNIRS brain activation (HbO) for Go/No-Go contrast by region of interest in farmworkers from the Zarcero County, Costa Rica (**excluding participants who had outliers in errors of commission**).

Contrast	Hemisphere	Position	***Urinary pesticide biomarkers***							
			**Insecticides**			**Fungicides**			**Herbicides**	
			**TCPy**	**3-PBA**	**DCCA**	**ETU**	**PTU**	**TEB-OH**	**2,4-D**	**GLY**
No-Go vs. Go (*n* = 47)	L	Inferior frontal pole	-0.46 (-2.51, 1.59)	-2.08 (-4.59, 0.44)	-1.45 (-3.95, 1.05)	0.64 (-1.37, 2.66)	0.15 (-1.75, 2.06)	-0.09 (-1.93, 1.74)	0.16 (-2.23, 2.54)	0.97 (-1.82, 3.77)
		Superior frontal pole	-1.45 (-3.81, 0.91)	-3.58 (-6.40, -0.77)*	-3.09 (-5.90, -0.28)*	-0.59 (-2.94, 1.77)	-1.51 (-3.69, 0.66)	-0.63 (-2.77, 1.51)	-0.06 (-2.85, 2.73)	1.29 (-1.96, 4.55)
		Broca/Broadmann	-0.71 (-2.93, 1.51)	-2.26 (-4.98, 0.47)	-1.89 (-4.58, 0.81)	0.34 (-1.86, 2.53)	-0.07 (-2.14, 1.99)	-0.93 (-2.91, 1.04)	0.53 (-2.06, 3.11)	0.78 (-2.25, 3.82)
		Dorsolateral prefrontal	-1.25 (-3.47, 0.98)	-2.63 (-5.35, 0.10)	-2.01 (-4.73, 0.70)	0.29 (-1.93, 2.51)	-0.62 (-2.70, 1.46)	0.30 (-1.71, 2.32)	-0.79 (-3.40, 1.82)	0.81 (-2.26, 3.89)
	R	Inferior frontal pole	-0.52 (-2.04, 1.00)	-0.53 (-2.44, 1.39)	-0.41 (-2.29, 1.47)	0.13 (-1.38, 1.63)	0.22 (-1.19, 1.64)	0.30 (-1.06, 1.66)	-0.63 (-2.40, 1.13)	1.69 (-0.33, 3.71)
		Superior frontal pole	-1.03 (-2.87, 0.80)	-1.63 (-3.93, 0.66)	-1.22 (-3.49, 1.05)	-0.18 (-2.02, 1.65)	-0.95 (-2.65, 0.75)	-0.11 (-1.77, 1.55)	-0.63 (-2.78, 1.52)	0.13 (-2.41, 2.67)
		Broca/Broadmann	-1.09 (-3.00, 0.82)	-0.87 (-3.30, 1.56)	-0.95 (-3.33, 1.43)	-0.31 (-2.22, 1.60)	-0.54 (-2.33, 1.25)	-0.13 (-1.87, 1.60)	-0.61 (-2.85, 1.64)	1.30 (-1.32, 3.92)
		Dorsolateral prefrontal	-1.84 (-3.70, 0.01)*	-1.22 (-3.64, 1.19)	-1.23 (-3.59, 1.13)	-1.60 (-3.44, 0.25)	-1.86 (-3.56, -0.16)*	-0.83 (-2.54, 0.88)	-1.35 (-3.56, 0.87)	-0.85 (-3.49, 1.78)

Abbreviations: fNIRS, functional Near-Infrared Spectroscopy; n, number of participants; L, left; R, right; ETU, ethylenethiourea; PTU, propylenethiourea; TCPy, 3,5,6-trichloro-2-pyridinol; 3-PBA, 3-phenoxybenzoic acid; DCCA, 3-(2,2-dichlorovinyl)-2,2-dimethylcyclopropanecarboxylic acid; 2,4-D, 2,4-dichlorophenoxyacetic acid; TEB-OH, hydroxy-tebuconazole; GLY, glyphosate.
Models adjusted for age (continuous variable) and education level (≤6th grade, 7-11th grade).
*non-FDR corrected p<0.05, † FDR-corrected p<0.05.

Table S14. Adjusted associations [β (95% CI)] for a two-fold increase in urinary pesticide biomarker (specific gravity-adjusted) concentrations (imputed) with fNIRS brain activation (HbO) for Wisconsin Card Sort Test contrast by region of interest in farmworkers from the Zarcero County, Costa Rica (**excluding participants who had outliers in matching block perseverative errors**).

Contrast	Hemisphere	Position	***Urinary pesticide biomarkers***							
			**Insecticides**			**Fungicides**			**Herbicides**	
			**TCPy**	**3-PBA**	**DCCA**	**ETU**	**PTU**	**TEB-OH**	**2,4-D**	**GLY**
Matching vs. control (*n* = 35)	L	Inferior frontal pole	-1.22 (-4.77, 2.32)	-1.33 (-6.02, 3.37)	-2.39 (-6.89, 2.12)	-1.78 (-5.33, 1.77)	-2.41 (-6.18, 1.35)	-1.53 (-5.08, 2.02)	-0.07 (-4.00, 3.85)	-1.71 (-6.41, 2.99)
		Superior frontal pole	-1.03 (-4.08, 2.02)	-1.70 (-5.71, 2.31)	-1.98 (-5.86, 1.90)	-1.78 (-4.81, 1.25)	-1.42 (-4.70, 1.87)	0.10 (-2.99, 3.19)	0.09 (-3.29, 3.46)	-1.81 (-5.84, 2.22)
		Broca/Broadmann	-0.16 (-3.66, 3.33)	-1.26 (-5.85, 3.33)	-1.93 (-6.37, 2.50)	-2.44 (-5.86, 0.97)	-3.95 (-7.45, -0.46)*	-1.43 (-4.90, 2.05)	0.29 (-3.55, 4.13)	-2.62 (-7.16, 1.92)
		Dorsolateral prefrontal	0.23 (-2.79, 3.25)	-2.32 (-6.22, 1.58)	-2.16 (-5.96, 1.64)	-2.16 (-5.10, 0.79)	-2.10 (-5.28, 1.08)	-0.44 (-3.47, 2.59)	-0.25 (-3.57, 3.07)	-2.07 (-6.01, 1.87)
	R	Inferior frontal pole	-1.30 (-4.72, 2.11)	-1.25 (-5.78, 3.27)	-2.07 (-6.43, 2.29)	-1.38 (-4.82, 2.06)	-1.63 (-5.31, 2.06)	-1.02 (-4.47, 2.42)	0.04 (-3.75, 3.83)	-0.79 (-5.36, 3.78)
		Superior frontal pole	-0.67 (-3.75, 2.41)	-0.67 (-4.75, 3.40)	-1.05 (-5.00, 2.90)	0.12 (-3.00, 3.24)	-1.24 (-4.56, 2.07)	0.55 (-2.55, 3.65)	0.47 (-2.92, 3.86)	0.21 (-3.90, 4.31)
		Broca/Broadmann	0.59 (-2.66, 3.83)	0.01 (-4.28, 4.30)	-1.02 (-5.18, 3.14)	-0.82 (-4.09, 2.44)	-0.86 (-4.36, 2.64)	-0.51 (-3.77, 2.75)	0.43 (-3.13, 4.00)	0.67 (-3.63, 4.98)
		Dorsolateral prefrontal	-0.75 (-3.58, 2.09)	-0.41 (-4.17, 3.35)	-1.08 (-4.72, 2.55)	-0.70 (-3.56, 2.16)	-1.77 (-4.78, 1.25)	0.09 (-2.78, 2.95)	1.15 (-1.95, 4.25)	-1.72 (-5.45, 2.01)

Abbreviations: fNIRS, functional Near-Infrared Spectroscopy; n, number of participants; L, left; R, right; ETU, ethylenethiourea; PTU, propylenethiourea; TCPy, 3,5,6-trichloro-2-pyridinol; 3-PBA, 3-phenoxybenzoic acid; DCCA, 3-(2,2-dichlorovinyl)-2,2-dimethylcyclopropanecarboxylic acid; 2,4-D, 2,4-dichlorophenoxyacetic acid; TEB-OH, hydroxy-tebuconazole; GLY, glyphosate.
Models adjusted for age (continuous variable) and education level (≤6th grade, 7-11th grade).
*non-FDR corrected p<0.05, † FDR-corrected p<0.05.

Table S15. Adjusted associations [β (95% CI)] for a two-fold increase in urinary pesticide biomarker (specific gravity-adjusted) concentrations (imputed) with fNIRS brain activation (HbO) by task and region of interest in farmworkers from the Zarcero County, Costa Rica (**adjusting for poverty status (imputed) in models**).

Contrast	Hemisphere	Position	***Urinary pesticide biomarkers***							
			**Insecticides**			**Fungicides**			**Herbicides**	
			**TCPy**	**3-PBA**	**DCCA**	**ETU**	**PTU**	**TEB-OH**	**2,4-D**	**GLY**
Encoding vs. recall (Sternberg test) (n = 48)	L	Inferior frontal pole	-1.95 (-3.81, -0.10)*	-2.04 (-4.25, 0.18)	-1.73 (-3.99, 0.52)	-0.61 (-2.57, 1.36)	0.31 (-1.56, 2.18)	-1.66 (-3.33, 0.01)*	-0.78 (-2.99, 1.43)	0.08 (-2.56, 2.72)
		Superior frontal pole	-1.77 (-3.34, -0.19)*	-2.32 (-4.14, -0.49)*	-2.25 (-4.10, -0.40)*	-0.59 (-2.26, 1.09)	-0.01 (-1.61, 1.58)	-1.03 (-2.48, 0.43)	-1.31 (-3.16, 0.54)	1.05 (-1.18, 3.28)
		Broca/Broadmann	-1.21 (-2.96, 0.54)	-1.80 (-3.83, 0.23)	-1.35 (-3.43, 0.73)	-0.02 (-1.82, 1.79)	1.02 (-0.67, 2.70)	-1.07 (-2.64, 0.49)	-0.65 (-2.68, 1.38)	0.08 (-2.34, 2.51)
		Dorsolateral prefrontal	-2.42 (-4.11, -0.74)*†	-3.09 (-5.04, -1.14)*†	-3.03 (-5.01, -1.05)*†	-1.16 (-2.99, 0.67)	-0.25 (-2.01, 1.51)	-1.55 (-3.12, 0.03)*	-1.32 (-3.38, 0.74)	0.39 (-2.10, 2.88)
	R	Inferior frontal pole	-0.78 (-2.80, 1.24)	-2.40 (-4.68, -0.12)*	-2.22 (-4.54, 0.10)	-0.27 (-2.32, 1.79)	0.87 (-1.06, 2.81)	-2.00 (-3.72, -0.29)*	0.05 (-2.27, 2.37)	-0.12 (-2.87, 2.63)
		Superior frontal pole	-1.10 (-2.75, 0.55)	-1.91 (-3.80, -0.02)*	-1.98 (-3.88, -0.08)*	-0.07 (-1.77, 1.63)	0.37 (-1.24, 1.97)	-1.24 (-2.69, 0.21)	-0.72 (-2.62, 1.18)	-0.03 (-2.30, 2.25)
		Broca/Broadmann	-0.68 (-2.43, 1.08)	-2.25 (-4.22, -0.28)*	-2.09 (-4.09, -0.09)*	-0.11 (-1.90, 1.68)	0.41 (-1.28, 2.10)	-1.28 (-2.81, 0.25)	0.04 (-1.98, 2.06)	-0.38 (-2.78, 2.01)
		Dorsolateral prefrontal	-1.02 (-2.94, 0.91)	-2.27 (-4.45, -0.08)*	-1.95 (-4.19, 0.28)	-0.44 (-2.41, 1.52)	0.77 (-1.08, 2.62)	-1.31 (-3.00, 0.38)	-0.51 (-2.72, 1.71)	0.37 (-2.26, 3.00)
No-Go vs. Go (n = 48)	L	Inferior frontal pole	-0.02 (-2.12, 2.08)	-1.45 (-3.88, 0.99)	-1.04 (-3.52, 1.43)	1.12 (-0.97, 3.22)	0.45 (-1.56, 2.46)	0.04 (-1.84, 1.92)	0.23 (-2.17, 2.62)	1.08 (-1.74, 3.91)
		Superior frontal pole	-0.69 (-3.17, 1.79)	-2.40 (-5.24, 0.43)	-2.31 (-5.17, 0.56)	0.09 (-2.43, 2.60)	-1.23 (-3.58, 1.13)	-0.44 (-2.66, 1.78)	0.07 (-2.76, 2.91)	1.43 (-1.91, 4.77)
		Broca/Broadmann	-0.50 (-2.77, 1.78)	-1.75 (-4.39, 0.89)	-1.59 (-4.26, 1.08)	0.56 (-1.74, 2.86)	0.01 (-2.18, 2.20)	-0.93 (-2.95, 1.09)	0.57 (-2.03, 3.17)	0.79 (-2.29, 3.87)
		Dorsolateral prefrontal	-0.76 (-3.05, 1.53)	-1.90 (-4.55, 0.75)	-1.54 (-4.23, 1.15)	0.83 (-1.48, 3.14)	-0.34 (-2.54, 1.86)	0.49 (-1.56, 2.54)	-0.71 (-3.32, 1.91)	0.96 (-2.14, 4.06)
	R	Inferior frontal pole	0.11 (-1.55, 1.76)	0.32 (-1.63, 2.27)	0.20 (-1.76, 2.16)	0.68 (-0.98, 2.33)	0.52 (-1.06, 2.10)	0.43 (-1.04, 1.90)	-0.52 (-2.40, 1.36)	1.71 (-0.46, 3.89)
		Superior frontal pole	-0.43 (-2.42, 1.56)	-0.66 (-3.00, 1.68)	-0.55 (-2.91, 1.81)	0.32 (-1.70, 2.33)	-0.82 (-2.71, 1.08)	-0.02 (-1.80, 1.76)	-0.52 (-2.78, 1.75)	0.09 (-2.61, 2.78)
		Broca/Broadmann	-0.91 (-2.87, 1.04)	-0.62 (-2.94, 1.70)	-0.77 (-3.10, 1.57)	-0.12 (-2.12, 1.87)	-0.43 (-2.32, 1.46)	-0.05 (-1.82, 1.71)	-0.57 (-2.82, 1.67)	1.40 (-1.24, 4.04)
		Dorsolateral prefrontal	-1.42 (-3.34, 0.49)	-0.72 (-3.02, 1.59)	-0.85 (-3.17, 1.46)	-1.26 (-3.21, 0.68)	-1.71 (-3.51, 0.10)	-0.68 (-2.42, 1.06)	-1.27 (-3.48, 0.93)	-0.71 (-3.36, 1.94)
Matching vs. control	L	Inferior frontal pole	-1.66 (-4.85, 1.52)	-1.76 (-5.83, 2.31)	-2.88 (-6.94, 1.19)	-1.87 (-5.34, 1.61)	-1.42 (-4.89, 2.05)	-1.33 (-4.70, 2.04)	0.10 (-3.58, 3.78)	-1.37 (-5.76, 3.02)

(Wisconsin Card Sort test) (*n* = 41)		Superior frontal pole	-1.52<br>(-4.26, 1.22)	-2.17<br>(-5.65, 1.30)	-2.54<br>(-6.05, 0.96)	-2.30<br>(-5.25, 0.65)	-0.97<br>(-3.98, 2.04)	0.05<br>(-2.88, 2.99)	0.03<br>(-3.14, 3.20)	-1.72<br>(-5.49, 2.04)
		Broca/Broadmann	-0.73<br>(-3.92, 2.45)	-1.28<br>(-5.32, 2.77)	-2.11<br>(-6.18, 1.95)	-3.21<br>(-6.53, 0.10)	-3.02<br>(-6.33, 0.29)	-1.29<br>(-4.62, 2.04)	0.53<br>(-3.09, 4.16)	-2.31<br>(-6.60, 1.98)
		Dorsolateral prefrontal	-0.52<br>(-3.24, 2.21)	-2.48<br>(-5.85, 0.89)	-2.56<br>(-5.98, 0.86)	-2.86<br>(-5.68, -0.04)*	-1.92<br>(-4.80, 0.97)	-0.47<br>(-3.34, 2.40)	-0.33<br>(-3.43, 2.77)	-1.92<br>(-5.59, 1.75)
	R	Inferior frontal pole	-1.32<br>(-4.35, 1.71)	-1.41<br>(-5.28, 2.47)	-2.30<br>(-6.19, 1.58)	-1.27<br>(-4.60, 2.05)	-1.33<br>(-4.62, 1.97)	-0.84<br>(-4.05, 2.37)	0.16<br>(-3.33, 3.65)	-0.42<br>(-4.61, 3.76)
		Superior frontal pole	-0.63<br>(-3.44, 2.18)	-1.11<br>(-4.68, 2.45)	-1.52<br>(-5.12, 2.08)	0.18<br>(-2.90, 3.25)	-1.36<br>(-4.38, 1.66)	0.60<br>(-2.35, 3.56)	0.39<br>(-2.81, 3.59)	0.43<br>(-3.41, 4.27)
		Broca/Broadmann	0.06<br>(-2.85, 2.98)	-0.52<br>(-4.23, 3.19)	-1.47<br>(-5.20, 2.27)	-1.05<br>(-4.21, 2.12)	-0.24<br>(-3.41, 2.92)	-0.34<br>(-3.41, 2.72)	0.45<br>(-2.87, 3.76)	0.74<br>(-3.24, 4.71)
		Dorsolateral prefrontal	-0.77<br>(-3.34, 1.81)	-0.62<br>(-3.91, 2.66)	-1.37<br>(-4.69, 1.94)	-0.67<br>(-3.49, 2.14)	-1.83<br>(-4.57, 0.91)	0.22<br>(-2.50, 2.94)	1.34<br>(-1.57, 4.25)	-1.14<br>(-4.65, 2.38)

Abbreviations: fNIRS, functional Near-Infrared Spectroscopy; n, number of participants; L, left; R, right; ETU, ethylenethiourea; PTU, propylenethiourea; TCPy, 3,5,6-trichloro-2-pyridinol; 3-PBA, 3-phenoxybenzoic acid; DCCA, 3-(2,2-dichlorovinyl)-2,2-dimethylcyclopropanecarboxylic acid; 2,4-D, 2,4-dichlorophenoxyacetic acid; TEB-OH, hydroxy-tebuconazole; GLY, glyphosate.

Models adjusted for age (continuous variable), education level (≤6th grade, 7-11th grade), and poverty status (≤poverty line, >poverty line).

*non-FDR corrected p<0.05, † FDR-corrected p<0.05.

Table S16. Adjusted associations [β (95% CI)] for a two-fold increase in urinary pesticide biomarker (specific gravity-adjusted) concentrations (imputed) with fNIRS brain activation (HbO) by task and region of interest in farmworkers from the Zarcero County, Costa Rica (**adjusting for ever used a computer or played videogames (imputed) in models**).

Contrast	Hemisphere	Position	***Urinary pesticide biomarkers***							
			**Insecticides**			**Fungicides**			**Herbicides**	
			**TCPy**	**3-PBA**	**DCCA**	**ETU**	**PTU**	**TEB-OH**	**2,4-D**	**GLY**
Encoding vs. recall (Sternberg test) (*n* = 48)	L	Inferior frontal pole	-1.85 (-3.64, -0.07)*	-2.01 (-4.22, 0.21)	-1.69 (-3.95, 0.57)	-0.58 (-2.45, 1.29)	0.31 (-1.49, 2.11)	-1.62 (-3.33, 0.08)	-0.94 (-3.18, 1.29)	-0.03 (-2.63, 2.58)
		Superior frontal pole	-1.64 (-3.15, -0.12)*	-2.29 (-4.12, -0.46)*	-2.22 (-4.08, -0.36)*	-0.52 (-2.12, 1.07)	0.03 (-1.51, 1.57)	-0.99 (-2.48, 0.49)	-1.43 (-3.30, 0.44)	0.99 (-1.22, 3.19)
		Broca/Broadmann	-1.19 (-2.87, 0.49)	-1.79 (-3.83, 0.24)	-1.34 (-3.43, 0.75)	-0.08 (-1.80, 1.65)	0.92 (-0.71, 2.56)	-1.08 (-2.67, 0.52)	-0.77 (-2.82, 1.29)	-0.02 (-2.41, 2.37)
		Dorsolateral prefrontal	-2.31 (-3.93, -0.68)*†	-3.12 (-5.07, -1.16)*†	-3.07 (-5.06, -1.09)*†	-1.13 (-2.88, 0.62)	-0.30 (-2.01, 1.41)	-1.65 (-3.25, -0.05)*	-1.37 (-3.46, 0.72)	0.34 (-2.13, 2.80)
	R	Inferior frontal pole	-0.74 (-2.68, 1.20)	-2.37 (-4.65, -0.09)*	-2.19 (-4.51, 0.14)	-0.25 (-2.20, 1.71)	0.85 (-1.01, 2.72)	-2.00 (-3.75, -0.25)*	-0.06 (-2.41, 2.29)	-0.19 (-2.91, 2.53)
		Superior frontal pole	-1.05 (-2.64, 0.53)	-1.92 (-3.81, -0.03)*	-2.00 (-3.91, -0.10)*	-0.10 (-1.72, 1.52)	0.31 (-1.25, 1.87)	-1.30 (-2.78, 0.18)	-0.76 (-2.69, 1.17)	-0.06 (-2.31, 2.19)
		Broca/Broadmann	-0.73 (-2.42, 0.96)	-2.27 (-4.24, -0.30)*	-2.12 (-4.13, -0.11)*	-0.21 (-1.92, 1.50)	0.29 (-1.35, 1.94)	-1.35 (-2.92, 0.21)	-0.02 (-2.08, 2.03)	-0.48 (-2.85, 1.89)
		Dorsolateral prefrontal	-1.10 (-2.94, 0.74)	-2.25 (-4.44, -0.06)*	-1.93 (-4.16, 0.31)	-0.55 (-2.42, 1.32)	0.64 (-1.15, 2.43)	-1.28 (-3.01, 0.45)	-0.74 (-2.98, 1.50)	0.13 (-2.47, 2.73)
No-Go vs. Go (*n* = 48)	L	Inferior frontal pole	-0.14 (-2.14, 1.85)	-1.38 (-3.78, 1.03)	-0.94 (-3.39, 1.51)	0.94 (-1.04, 2.92)	0.44 (-1.48, 2.36)	0.26 (-1.63, 2.15)	-0.09 (-2.48, 2.31)	0.80 (-1.97, 3.56)
		Superior frontal pole	-0.75 (-2.93, 1.43)	-2.16 (-4.77, 0.45)	-1.96 (-4.61, 0.69)	0.11 (-2.10, 2.31)	-0.89 (-2.99, 1.21)	0.21 (-1.87, 2.29)	-0.69 (-3.32, 1.94)	0.90 (-2.15, 3.94)
		Broca/Broadmann	-0.43 (-2.59, 1.73)	-1.63 (-4.23, 0.97)	-1.43 (-4.07, 1.21)	0.61 (-1.56, 2.77)	0.19 (-1.89, 2.27)	-0.66 (-2.70, 1.38)	0.30 (-2.30, 2.89)	0.63 (-2.37, 3.63)
		Dorsolateral prefrontal	-0.84 (-2.98, 1.29)	-1.78 (-4.37, 0.80)	-1.38 (-4.01, 1.26)	0.68 (-1.47, 2.84)	-0.25 (-2.33, 1.82)	0.85 (-1.18, 2.87)	-1.18 (-3.75, 1.38)	0.59 (-2.40, 3.58)
	R	Inferior frontal pole	-0.03 (-1.61, 1.55)	0.36 (-1.57, 2.29)	0.27 (-1.68, 2.22)	0.51 (-1.06, 2.09)	0.46 (-1.05, 1.97)	0.59 (-0.90, 2.07)	-0.78 (-2.66, 1.10)	1.45 (-0.69, 3.60)
		Superior frontal pole	-0.44 (-2.27, 1.40)	-0.51 (-2.76, 1.74)	-0.35 (-2.63, 1.93)	0.33 (-1.52, 2.17)	-0.60 (-2.36, 1.17)	0.37 (-1.37, 2.11)	-0.95 (-3.15, 1.24)	-0.17 (-2.73, 2.39)
		Broca/Broadmann	-0.87 (-2.69, 0.96)	-0.50 (-2.76, 1.76)	-0.60 (-2.87, 1.68)	-0.07 (-1.92, 1.79)	-0.26 (-2.03, 1.52)	0.27 (-1.48, 2.02)	-0.94 (-3.14, 1.26)	1.16 (-1.38, 3.71)
		Dorsolateral prefrontal	-1.54 (-3.37, 0.29)	-0.71 (-3.02, 1.60)	-0.83 (-3.16, 1.50)	-1.36 (-3.22, 0.49)	-1.73 (-3.48, 0.01)*	-0.64 (-2.42, 1.14)	-1.56 (-3.78, 0.66)	-0.99 (-3.60, 1.63)
Matching vs. control	L	Inferior frontal pole	-1.66 (-4.39, 1.08)	-1.58 (-5.18, 2.02)	-2.38 (-6.00, 1.25)	-1.20 (-4.17, 1.77)	-0.54 (-3.59, 2.50)	-0.42 (-3.47, 2.62)	-0.85 (-4.09, 2.39)	-2.01 (-5.84, 1.83)

(Wisconsin Card Sort test) (*n* = 41)		Superior frontal pole	-1.29 (-3.74, 1.16)	-1.99 (-5.17, 1.19)	-2.13 (-5.37, 1.10)	-1.40 (-4.03, 1.24)	-0.02 (-2.75, 2.70)	0.92 (-1.79, 3.62)	-0.50 (-3.40, 2.41)	-2.03 (-5.44, 1.38)
		Broca/Broadmann	-0.60 (-3.40, 2.20)	-1.07 (-4.72, 2.57)	-1.62 (-5.32, 2.08)	-2.22 (-5.15, 0.70)	-1.93 (-4.93, 1.07)	-0.34 (-3.40, 2.72)	-0.19 (-3.47, 3.09)	-2.75 (-6.56, 1.05)
		Dorsolateral prefrontal	-0.29 (-2.87, 2.29)	-2.34 (-5.62, 0.94)	-2.29 (-5.64, 1.06)	-2.02 (-4.71, 0.68)	-1.14 (-3.93, 1.66)	0.14 (-2.68, 2.96)	-0.57 (-3.58, 2.44)	-2.03 (-5.57, 1.52)
	R	Inferior frontal pole	-1.27 (-3.94, 1.41)	-1.24 (-4.75, 2.27)	-1.86 (-5.41, 1.68)	-0.64 (-3.54, 2.26)	-0.50 (-3.45, 2.46)	-0.01 (-2.97, 2.94)	-0.61 (-3.76, 2.54)	-0.94 (-4.70, 2.83)
		Superior frontal pole	-0.48 (-3.01, 2.05)	-0.95 (-4.25, 2.35)	-1.13 (-4.49, 2.23)	0.80 (-1.92, 3.51)	-0.49 (-3.26, 2.28)	1.41 (-1.32, 4.14)	-0.13 (-3.10, 2.83)	0.10 (-3.44, 3.64)
		Broca/Broadmann	0.08 (-2.49, 2.65)	-0.35 (-3.70, 3.00)	-1.03 (-4.44, 2.37)	-0.40 (-3.16, 2.35)	0.56 (-2.24, 3.36)	0.48 (-2.32, 3.28)	-0.26 (-3.26, 2.74)	0.26 (-3.32, 3.84)
		Dorsolateral prefrontal	-0.70 (-3.02, 1.62)	-0.49 (-3.53, 2.55)	-1.03 (-4.11, 2.06)	-0.17 (-2.67, 2.34)	-1.13 (-3.65, 1.40)	0.89 (-1.63, 3.42)	0.79 (-1.92, 3.50)	-1.49 (-4.71, 1.72)

Abbreviations: fNIRS, functional Near-Infrared Spectroscopy; n, number of participants; L, left; R, right; ETU, ethylenethiourea; PTU, propylenethiourea; TCPy, 3,5,6-trichloro-2-pyridinol; 3-PBA, 3-phenoxybenzoic acid; DCCA, 3-(2,2-dichlorovinyl)-2,2-dimethylcyclopropanecarboxylic acid; 2,4-D, 2,4-dichlorophenoxyacetic acid; TEB-OH, hydroxy-tebuconazole; GLY, glyphosate.

Models adjusted for age (continuous variable), education level (≤6th grade, 7-11th grade), and computer literacy (yes, no).

*non-FDR corrected p<0.05, † FDR-corrected p<0.05.

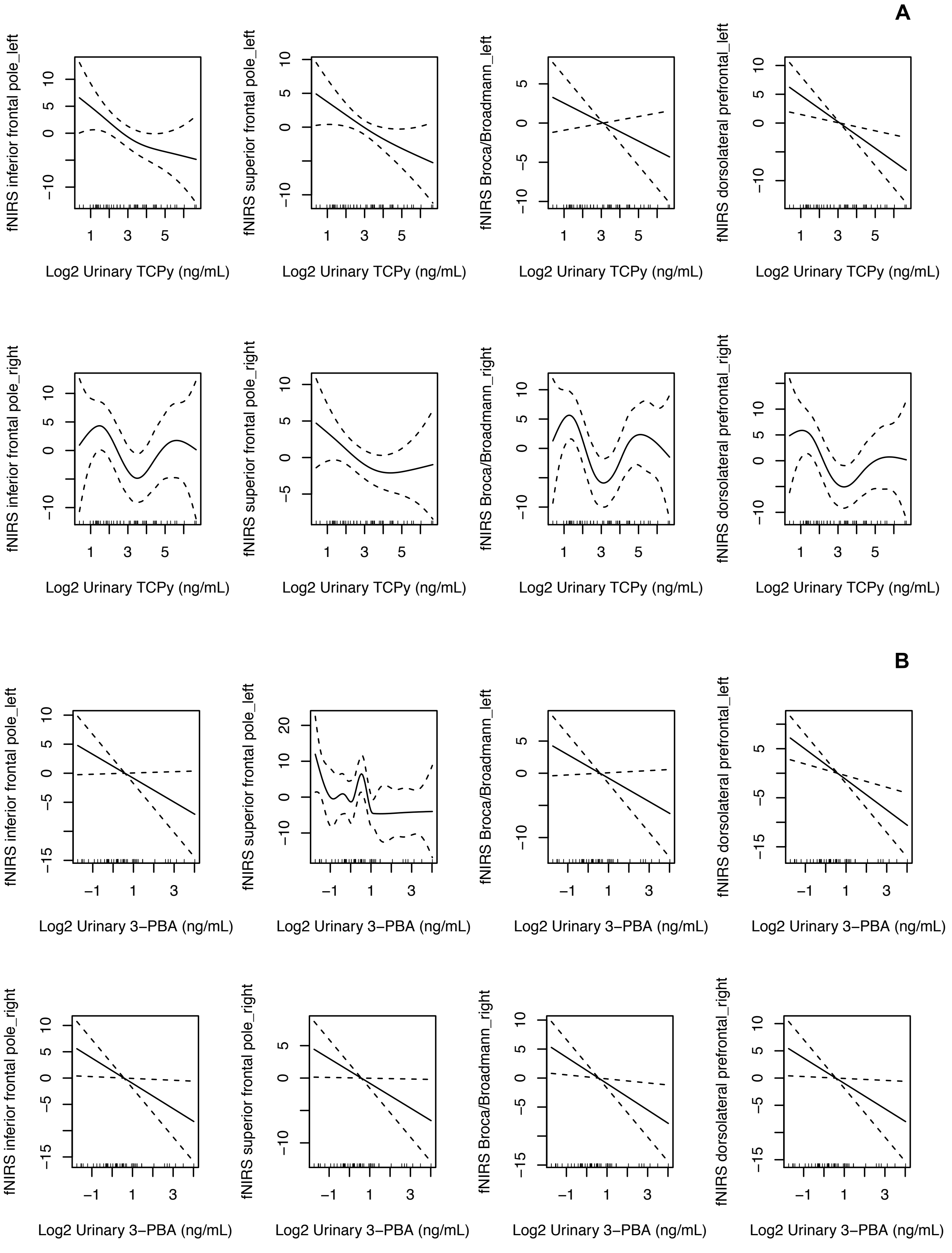

A
fNIRS inferior frontal pole_left
fNIRS superior frontal pole_left
fNIRS Broca/Broadmann_left
fNIRS dorsolateral prefrontal_left
fNIRS inferior frontal pole_right
fNIRS superior frontal pole_right
fNIRS Broca/Broadmann_right
fNIRS dorsolateral prefrontal_right
Log2 Urinary TCPy (ng/mL)
B
fNIRS inferior frontal pole_left
fNIRS superior frontal pole_left
fNIRS Broca/Broadmann_left
fNIRS dorsolateral prefrontal_left
fNIRS inferior frontal pole_right
fNIRS superior frontal pole_right
fNIRS Broca/Broadmann_right
fNIRS dorsolateral prefrontal_right
Log2 Urinary 3−PBA (ng/mL)

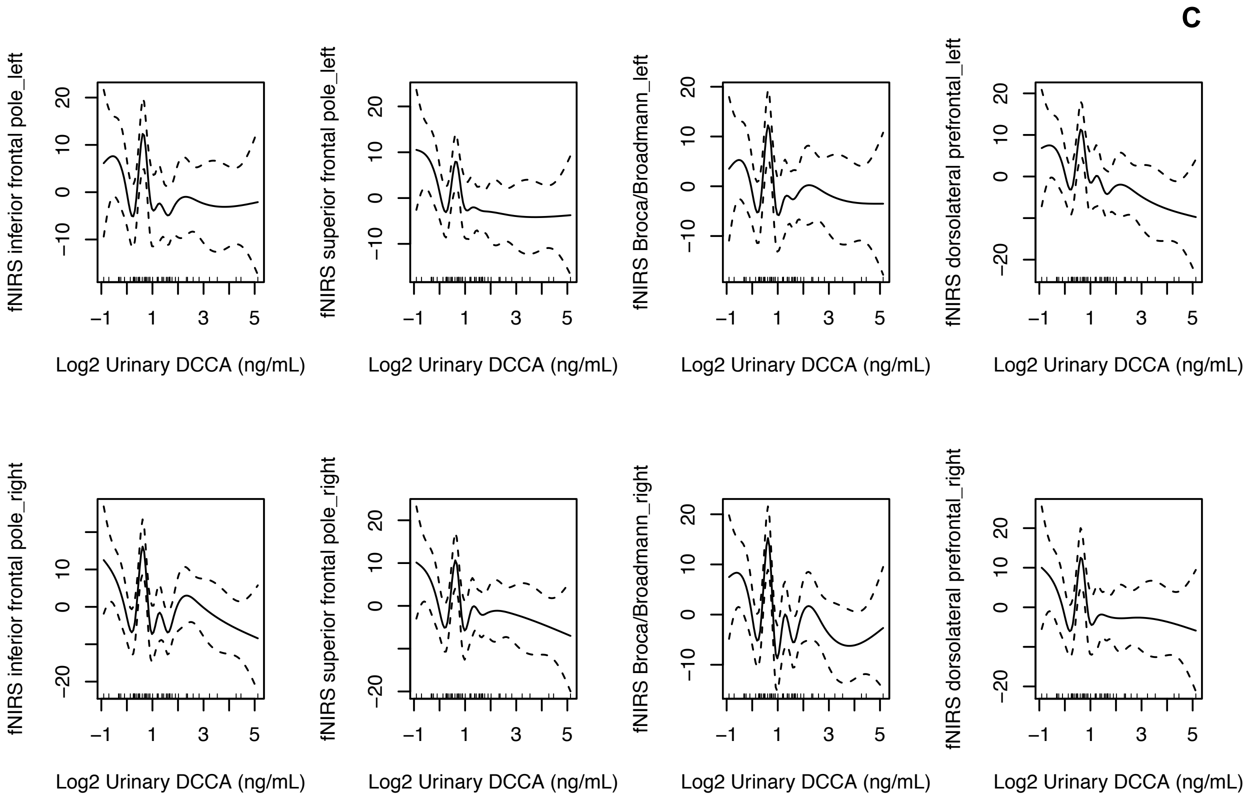

Figure S2. Smoothed associations of urinary pesticide metabolite (specific gravity-adjusted) concentrations with fNIRS brain activation (HbO) in farmworkers from the Zarcero County, Costa Rica. Generalized additive models were fit using penalized splines with smoothing parameters estimated by generalized cross-validation [*gam()* function in the R package *mgcv*]. Models were adjusted for age and education level. Log_2-transformed and specific-gravity adjusted urinary (A) TCPy; (B) 3-PBA; (C) DCCA. *Abbreviations*: TCPy, 3,5,6-trichloro-2-pyridinol; 3-PBA, 3-phenoxybenzoic acid; DCCA, 3-(2,2-dichlorovinyl)-2,2-dimethylcyclopropanecarboxylic acid.

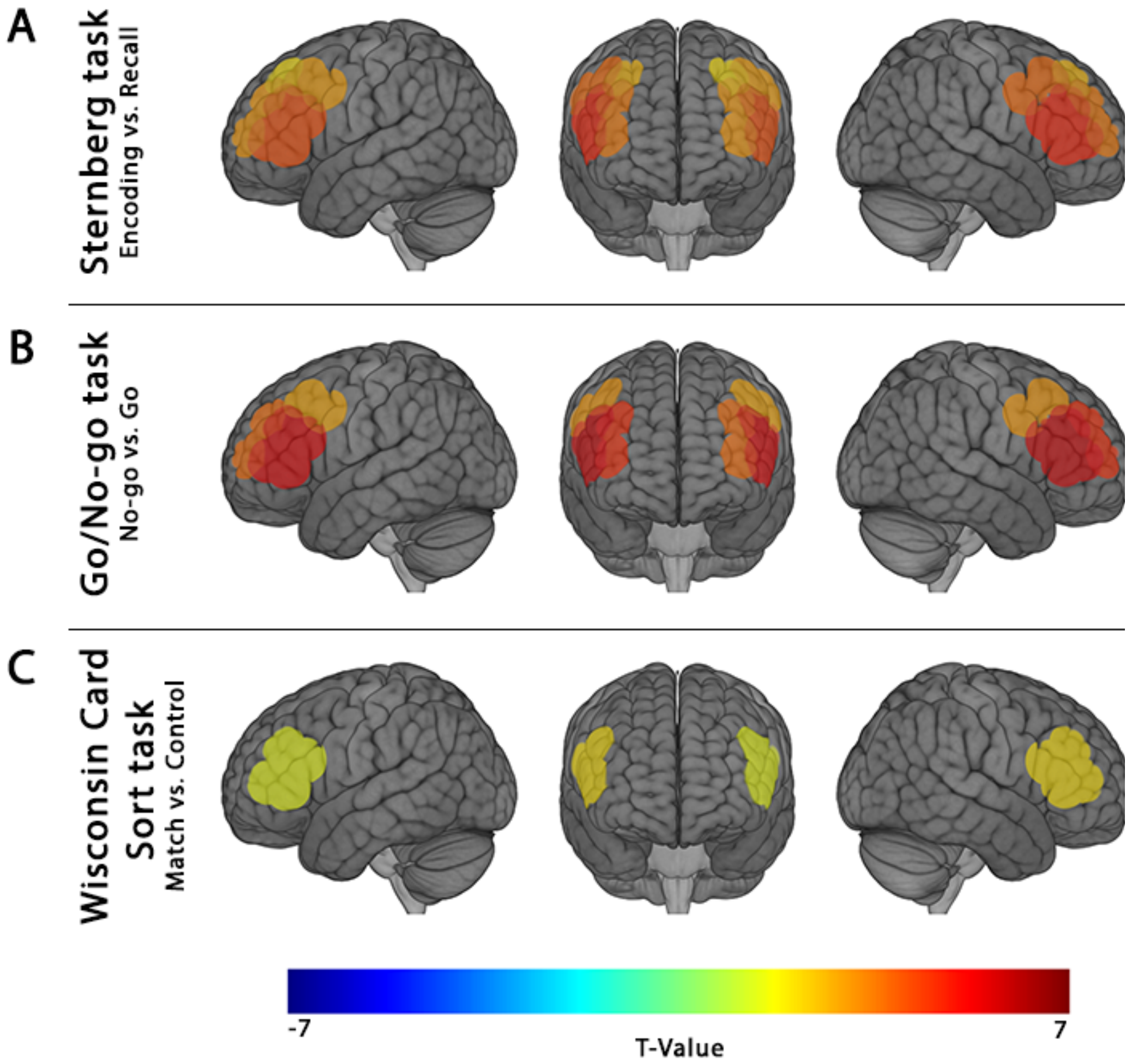

Figure S3. Cortical brain activation across the eight regions of interest in response to the (A) Sternberg working memory task (n = 48), (B) Go/No-Go task (n = 48), and (C) Wisconsin Card Sorting Test (n = 41) in farmworkers from Zarcero County, Costa Rica. Colors represent T-scores with warm colors indicating positive values and cool colors indicating negative values.

German External Quality Assessment Scheme

Intercomparison programme 64, 2019

for toxicological analyses in biological materials

Prof. Dr. med. H. Drexler

on behalf of the German Society for Occupational and Environmental Medicine .e.V

Henkestr. 9-11, D-91054 Erlangen

External Quality Control acc. to the Guidelines of the German Federal Medical Council

Teilnehmer: Arbets- och miljomedicin Syd, v 3 336
Medicon Village, Byggn 402A
Scheelevaegen 8
22185 Lund
Sweden

Certificate

valid until January 31, 2021

This is to certify you participated in the intercomparison programme 64 / 2019 for occupational / environmental medical - toxicological analyses. In accordance with the guidelines issued by the German Federal Medical Council (Bundesärztekammer) of September 19th, 2014 on implementation of intercomparison programmes in the medical field you have fullfilled the requirements for the following parameters:

Environmental medical field

Pb in blood
Cd in urine
Pt in urine
Cotinine in urine
MEHP in urine
MBzP in urine
Sr in urine
Mo in urine
Benzophenone-3 in urine

Cd in blood
Ni in urine
1-HP in urine
5-OH-MEHP in urine
PFOA in serum
As total in urine
Zn in urine
TCS in urine

Hg in blood
Hg in urine
3-PBA in urine
5-carboxy-MEPP in urine
PFOS in serum
Cu in urine
Trichloropyridinol in urine
Glyphosat in urine

Erlangen, 2020/01/28

Prof. Dr. med. H. Drexler

Prof. Dr. rer. nat. Th. Göen
